# Choosing T-cell sources determines CAR-T cell activity in neuroblastoma

**DOI:** 10.3389/fimmu.2024.1375833

**Published:** 2024-03-27

**Authors:** Lorena García-García, Elena G. Sánchez, Mariya Ivanova, Keren Pastora, Cristina Alcántara-Sánchez, Jorge García-Martínez, Beatriz Martín-Antonio, Manuel Ramírez, África González-Murillo

**Affiliations:** ^1^ Department of Pediatric Hematology and Oncology, Hospital Infantil Universitario Niño Jesús, Madrid, Spain; ^2^ Advanced Therapies Unit, Fundación Investigación Biomédica Hospital Infantil Universitario Niño Jesús, Madrid, Spain; ^3^ Department of Progenitor and Cell Therapy Research Group, La Princesa Institute of Health Research, Madrid, Spain; ^4^ Department of Experimental Hematology, Instituto de Investigación Sanitaria-Fundación Jiménez Díaz, Madrid, Spain

**Keywords:** immunotherapy, pediatric solid tumor, universal CAR-T cell, GD2, persistence, exhaustion

## Abstract

**Introduction:**

The clinical success of chimeric antigen receptor-modified T cells (CAR-T cells) for hematological malignancies has not been reproduced for solid tumors, partly due to the lack of cancer-type specific antigens. In this work, we used a novel combinatorial approach consisting of a versatile anti-FITC CAR-T effector cells plus an FITC-conjugated neuroblastoma (NB)-targeting linker, an FITC-conjugated monoclonal antibody (Dinutuximab) that recognizes GD2.

**Methods:**

We compared cord blood (CB), and CD45RA-enriched peripheral blood leukapheresis product (45RA) as allogeneic sources of T cells, using peripheral blood (PB) as a control to choose the best condition for anti-FITC CAR-T production. Cells were manufactured under two cytokine conditions (IL-2 *versus* IL-7+IL-15+IL-21) with or without CD3/CD28 stimulation. Immune phenotype, vector copy number, and genomic integrity of the final products were determined for cell characterization and quality control assessment. Functionality and antitumor capacity of CB/45RA-derived anti-FITC CAR-T cells were analyzed in co-culture with different anti-GD2-FITC labeled NB cell lines.

**Results:**

The IL-7+IL-15+IL-21 cocktail, in addition to co-stimulation signals, resulted in a favorable cell proliferation rate and maintained less differentiated immune phenotypes in both CB and 45RA T cells. Therefore, it was used for CAR-T cell manufacturing and further characterization. CB and CD45RA-derived anti-FITC CAR-T cells cultured with IL-7+IL-15+IL-21 retained a predominantly naïve phenotype compared with controls. In the presence of the NB-FITC targeting, CD4+ CB-derived anti-FITC CAR-T cells showed the highest values of co-stimulatory receptors OX40 and 4-1BB, and CD8+ CAR-T cells exhibited high levels of PD-1 and 4-1BB and low levels of TIM3 and OX40, compared with CAR-T cells form the other sources studied. CB-derived anti-FITC CAR-T cells released the highest amounts of cytokines (IFN-γ and TNF-α) into co-culture supernatants. The viability of NB target cells decreased to 30% when co-cultured with CB-derived CAR-T cells during 48h.

**Conclusion:**

CB and 45RA-derived T cells may be used as allogeneic sources of T cells to produce CAR-T cells. Moreover, ex vivo culture with IL-7+IL-15+IL-21 could favor CAR-T products with a longer persistence in the host. Our strategy may complement the current use of Dinutuximab in treating NB through its combination with a targeted CAR-T cell approach.

## 1 Introduction

Neuroblastoma (NB) is a neuroendocrine tumor that arises in the developing sympathetic nervous system, resulting in tumors in the adrenal glands and/or sympathetic ganglia. NB is the most common extracranial solid tumor in children, with 25–50 cases per million individuals (1), representing 8% of all pediatric cancers (2, 3). Despite the use of intense treatment with surgery, high-dose chemotherapy, and radiotherapy, only 45% of patients achieve 5-year event-free survival (4, 5). In the last decades, the development of immunotherapies has achieved remarkable progress in treating adult cancers. However, this development has not fundamentally changed the therapeutic approach to childhood cancer. The reason is that pediatric cancer differs from adult cancer in the lack of targetable alterations. Indeed, like most pediatric cancers, neuroblastoma manifests as lesions with low mutation burden and an absence of neoantigens targetable by the immune system (5). Likewise, long-term toxicities of potential treatments must be considered in pediatric cancers (6, 7).

Adoptive cell therapies encompass a spectrum of strategies based on ex vivo manipulation of immune cells to enhance their antitumor activity, which include tumor-infiltrating lymphocytes (TILs) and cells expressing transgenic T cell receptor (TCR), or chimeric antigen receptors (CAR). CAR T-cell therapy generally uses a single-chain variable fragment (scFv) derived from the variable fragment of the heavy and light chains of an antibody to target an extracellular antigen in an HLA-independent manner (8). A CAR is encoded by a single gene consisting of an scFv, a hinge or spacer, a transmembrane region, a signaling domain such as the CD3ζ chain from the TCR complex, and a co-stimulation domain (mainly 4-1BB and/or CD28) (9). Clinical experience with CAR T-cell therapy has been generally successful, mainly in hematological malignancies, with the development of anti-CD19 CAR treatments such as Kymriah, Yescarta, Breyanzi, and Tecartus, all approved by the U.S. Food and Drug Administration (FDA) for the treatment of B-cell malignancies.

Clinical results reported utilizing CARs to target adult or pediatric solid tumors have shown less efficacy than those in hematological tumors, in part due to the lack of targetable specific antigens and to the suppressive tumor microenvironment (TME) in solid tumors (6). The TME is composed of cell subsets that antagonize tumor-directed immune responses, such as myeloid-derived suppressor cells, tumor-associated macrophages or regulatory T cells (Tregs). Moreover, in terms of metabolism, these cells modulate the expression of indoleamine 2,3-dioxygenase (IDO) in dendritic cells, which leads to T cell exhaustion via essential amino acid depletion (10). Also, the lengthy culture period used to generate CAR-T cells and the use of autologous T cells from patients leads to T cell exhaustion, dysfunction, and low persistence in the host. In the case of NB, all CAR clinical trials have used GD2 or L1-CAM (CD171)-based CARs showing low persistence and efficacy (11, 12).

Classically, for CAR-T production, T lymphocytes from peripheral blood mononuclear cells (PBMCs) are activated through cross-linking antibodies (CD3 and/or CD28), transduced with gammaretroviral or lentiviral vectors encoding the CAR, and then expanded ex vivo using IL-2 (more recently also with IL-7+IL-15 (13–16), although IL-2 (17–20) is still the most used cytokine for ex vivo culture of lymphocytes). However, fundamental questions remain regarding the optimal cell source, target cell, expansion, and quality of therapeutic T cells used for transfer.

A critical factor influencing *in vivo* expansion is CAR-T cell phenotype. The phenotype of T cells that are transferred with viral vectors encoding CAR sequences and then infused into the patient plays an essential role in their persistence and cytolytic activity in the host. Early memory T cell subsets, including naïve T cells (T_N_), central memory T cells (T_CM_) and stem central memory T cells (T_SCM_) express CD45RA and have been proved to be the ones persisting in the host and showing functionality. Stem-like cells have self-renewal capacity and multipotency, are highly proliferative, and have enhanced antitumor activity in animal models when compared to other T cell subsets (21–23). Indeed, naïve T cells revealed higher *in vivo* engraftment, persistence, and xenoreactivity than central memory T cells in primary and secondary transplant recipients (24). In addition, cytokines impact the phenotype of T cells in culture. IL-7 drives the homeostatic expansion of naïve T cells and maintains the pool of central memory T cells (25). Using IL-7/IL-15 in culture produces more stem central memory T cells, which makes it a promising strategy to obtain CAR-T cells that kill and proliferate better in the body (26). Moreover, IL-21 has been shown to suppress the differentiation of CD8+ T cells into effector T cells, keeping them in a stem central memory -like state associated with high proliferative potential and long-term T cell survival (27–29).

In this study, we analyzed different strategies to improve the efficacy of CARs in solid tumors, focusing in the characterization of a new strategy to target Neuroblastoma, based on the combination of the monoclonal antibody (Dinutuximab) conjugated with FITC and anti-FITC CAR-T cells. In this system, we were interested in analyzing the proliferation of stem-like cells such as naïve T cells from different sources after expansion.

The 45RA fraction is obtained through a negative immunomagnetic selection to avoid naïve subpopulations in the product of T cell transplant from a sibling donor. However, in an autologous context it could be an important source of naïve T lymphocytes for protocols of adoptive cell transfer. Additionally, CB has some inherent features, such as rapid expansion of T cells, lower prevalence of graft-versus-host disease and higher graft versus tumor efficacy that make this hematopoietic stem and progenitor cell (HSPC) source more favorable than other HSPC sources (30–32). CB lymphocytes have been demonstrated to be suitable for adoptive cell transfer, taking into account their ability to maintain an early differentiation phenotype that could lead to long-term *in vivo* persistence after infusion (31–34). Accordingly, in our system we compared the function and phenotype of T cells obtained from different allogeneic sources such as PB, 45RA fraction after apheresis, and from CB, with the aim of enhancing the enrichment of the T cell populations with a more naïve/memory phenotype (CD45RA+) that may have better expansion and antitumor capacity *in vivo*.

The announcement on November 2023 by the FDA of serious risks of T-cell malignancies after autologous CAR-T targeting B-cell maturation antigen (BCMA) or CD19 highlights the importance of studying the safety of gene therapy products. Therefore, as a secondary objective of our work, we evaluated both aneuploidies, structural and copy number variants employing optical genome mapping technology, and the Vector Copy Number of the transgene by droplet digital PCR to evaluate the risk of genotoxicity in our system.

Here, we will present results of two main topics of research: (I) the study of proliferation, phenotype and functionality of T cells from different healthy donor sources to determine the best culture conditions in order to use them as allogeneic sources of T cell and (II) the study of phenotype and functionality of CAR-T cells derived from different sources in culture with the condition previously tested. In addition, we analyzed antitumor activity *in vitro* by the transduction of anti-FITC specific CAR construction.

## 2 Materials and methods

### 2.1 Isolation of T cells and cell lines

Mononuclear cells (MC) were harvested from different healthy donor sources such as peripheral blood (PB), cord blood (CB) and CD45RA+ T cells from leukapheresis (45RA). PB and 45RA T cells were provided by the Blood Bank Service of Hospital Infantil Universitario Niño Jesús. CB samples were provided from the Cord Blood Bank of Centro de Transfusión de la Comunidad de Madrid. The use of all these samples was approved by the Ethics Committee of Hospital Infantil Universitario Niño Jesús and Centro de Transfusión.

PBMCs and CBMCs were isolated by Ficoll-Hypaque (GE Healthcare) gradient separation. MC and 45RA T cells were cultured in “Complete Medium (CM)” composed of RPMI Medium 1640 (1x) + Glutamax (Gibco), supplemented with 10% human serum type AB (Sigma-Aldrich), 0.5% penicillin/streptomycin (10000Units/ml penicillin, 10000μg/ml streptomycin, Gibco), 2mM L-Glutamine (200mM, Gibco) and 25mM HEPES buffer solution (1M, Gibco).

In the first assays, cells were cultured with different combinations of cytokines: (I) 200IU/ml IL-2 (Proleukin Novartis) or (II) 10ng/ml (1,170IU/ml) IL-7 (Peprotech), 10ng/ml (287IU/ml) IL-15 (Peprotech) and 10ng/ml IL-21 (Peprotech) to compare which condition enhanced cell proliferation and their phenotype. In the later assays, IL-7+IL-15+IL-21 combination was used to produce CAR-T cells.

To study the functionality of CAR-T cells, we used different NB cell lines. LAN-1 and LAN-5 cell lines were purchased from Leibniz Institute DSMZ (German Collection of Microorganisms and Cell Cultures GmbH). SHSY5Y and SKNSH cell lines were provided by Dr. Javier Alonso from Instituto de Salud Carlos III (ISCIII), originally purchased from ATCC. LAN-1 cell line (ACC 655, DSMZ) was cultured in DMEM (Dulbecco’s Modified Eagle Medium) (1x) high glucose, GlutaMAX™ Supplement, pyruvate (Gibco), supplemented with 10% fetal bovine serum (FBS, Gibco) and 0.5% penicillin/streptomycin (10000Units/ml penicillin, 10000μg/ml streptomycin, Gibco). LAN-5 (ACC 673, DSMZ) and SHSY5Y (CRL-2266, ATCC) cell lines were cultured in RPMI Medium 1640 (1x) + Glutamax (Gibco), supplemented with 20% fetal bovine serum (FBS, Gibco) and 0.5% penicillin/streptomycin (10000Units/ml penicillin, 10000μg/ml streptomycin, Gibco). SKNSH (HTB-11, ATCC) cell line was cultured in RPMI Medium 1640 (1x) + Glutamax (Gibco), supplemented with 10% fetal bovine serum (FBS, Gibco) and 0.5% penicillin/streptomycin (10000Units/ml penicillin, 10000μg/ml streptomycin, Gibco).

To produce lentivirus supernatant stocks, 293T cell line was used and cultured in DMEM (Dulbecco’s Modified Eagle Medium) (1x) high glucose, GlutaMAX™ Supplement, pyruvate (Gibco), supplemented with 10% fetal bovine serum (FBS, Gibco) and 0.5% penicillin/streptomycin (10000Units/ml penicillin, 10000μg/ml streptomycin, Gibco).

All cell lines were regularly validated to be Mycoplasma free.

### 2.2 T cell proliferation assay

Mononuclear cells were activated with Dynabeads® Human T-Activator CD3/CD28 (Gibco) at bead-to-cell ratio of 1:2 24h after culture started. Other ratios (1:1 and 3:1) were tested in addition to the duration of the stimulus (short if beads were magnetically removed 48h after initiating T cell activation or long if beads were kept during all the experiment) (data shown in **Supplementary Figures**).

Cytokines and medium were replaced every 2-3 days, and viable cells were counted by Trypan Blue dye exclusion to maintain cellular concentration at 10^6^ cells/ml. The fold expansion was calculated using the viable cell number at each indicated time point divided by the viable cell number plated the previous day of expansion (1x10^6^ cells), everything multiplied by the number of cells plated on day 0.

### 2.3 Flow cytometry and antibodies

Cells were labeled with fluorescent antibodies (shown in **Table 1**) against human to study the phenotype of different subsets of T cells (shown in the **Table 2**), the early activation of T cells (CD25+ CD134+) or the exhaustion of T lymphocytes by measuring the levels of inhibitory receptors.

**Table 1:** Antibodies and isotypes used for flow cytometry.

FITC	TCRγδ (clone 11F2, BD Biosciences), CD25 (clone M-A251, BD Biosciences), PD1 (CD279) (clone EH12.2H7, BioLegend), CD56 (clone HCD56, BioLegend), mouse IgG1, κ (clone MOPC-21, Biolegend)
PE	TCRαβ (clone BW242/412, Miltenyi Biotec), CD122 (clone Mik-β3, BD Biosciences), CD4 (clone SK3, BioLegend), CD25 (clone M-A251, BD Biosciences)
PECy5	CD4 (clone SK3, BD Biosciences), OX40 (CD134) (clone ACT35, BD Biosciences), mouse IgG1, κ (clone X40, BD Bioscience)
PECy7	CD56 (clone NCAM16.2, BD Biosciences), CD3 (clone UCHT1, BioLegend), TIM3 (clone F38-2E2, BioLegend), IL-2 (clone MQ1-17H12, BioLegend), mouse IgG1, κ (clone MOPC-21, Biolegend)
APC	CD127 (clone HIL-7R-M21, BD Biosciences), 4-1BB (CD137) (clone 4B4-1, BioLegend), CD45RA (clone REA562, Miltenyi Biotec), mouse IgG1, κ (clone MOPC-21, Biolegend)
APCCy7	CD8 (clone SK1, BioLegend)
PB	CD3 (clone UCHT1, BD Biosciences), CCR7 (clone 150503, BD Biosciences), TNFα (clone Mab11, BioLegend), CD4 (clone SK3, BioLegend), mouse IgG1, κ (clone MOPC-21, Biolegend)
AC	CD45 (clone 2D1, BD Biosciences), CD45RA (clone HI100, BD Biosciences), IFNγ (clone B27, BD Biosciences), PD1 (CD279) (clone EH12.2H7, BioLegend), mouse IgG1, κ (clone MOPC-21, Biolegend)

**Table 2:** Cell populations studied by flow cytometry.

CELL TYPE	PHENOTYPE
Leucocytes	CD45+
T lymphocytes	CD45+ CD3+
TCRαβ	CD45+ CD3+ TCRαβ+
TCRγδ	CD45+ CD3+ TCRγδ+
Helper T lymphocytes	CD45+ CD3+ CD4+
Regulatory T lymphocytes	CD45+ CD3+ CD4+ CD25+ CD127-
CD4+ Naïve T cells	CD45+ CD3+ CD4+ CD45RA+ CCR7+
CD4+ Central memory T cells	CD45+ CD3+ CD4+ CD45RA- CCR7+
CD4+ Effector memory T cells	CD45+ CD3+ CD4+ CD45RA- CCR7-
CD4+ Terminal effector T cells	CD45+ CD3+ CD4+ CD45RA+ CCR7-
Cytotoxic T lymphocytes	CD45+ CD3+ CD8+
CD8+ Naïve T cells	CD45+ CD3+ CD8+ CD45RA+ CCR7+
CD8+ Central memory T cells	CD45+ CD3+ CD8+ CD45RA- CCR7+
CD8+ Effector memory T cells	CD45+ CD3+ CD8+ CD45RA- CCR7-
CD8+ Terminal effector T cells	CD45+ CD3+ CD8+ CD45RA+ CCR7-

Cell phenotype was studied in fresh samples and after 14 days of culture using the gating strategy for identifying T cell subsets shown in **Supplementary Figure S1**. In the case of CAR-T cell assays, the phenotype was analyzed 7 days after transduction. Staining protocol was performed at 4°C during 30 min and erythrocytes were lysed with quicklysis (Cytognos). Events were acquired using a BD FacsCanto II cytometer, and data were analyzed with FacsDiva software (BD) and FlowJo software version 10.6.1.

### 2.4 Intracellular cytokine staining

Intracellular cytokine expression was analyzed at the end of the experiment to study the functionality of T cells after proliferation. Cells were stimulated with a leukocyte activation cocktail (Intracellular Cytokine Staining Starter Kit, BD Biosciences), a ready-to-use polyclonal cell activation mixture containing a phorbol ester (Phorbol 12-Myristate 13-Acetate, PMA), a calcium ionophore (Ionomycin), and a protein transport inhibitor (BD GolgiPlug™, containing brefeldin A). Cells were stimulated during 4 hours at 37°C. Cells were stained with surface antibodies, washed, and fixed with BD IntraSure kit, and intracellular staining was performed with anti-IL-2, anti-IFN-γ and anti-TNF-α. Corresponding isotypes and blocking (Purified blocking antibody cocktail) antibodies were used as control.

Events were acquired using a BD FacsCanto II cytometer and data were analyzed by FacsDiva software (BD) and FlowJo software version 10.6.1.

### 2.5 CAR construction and lentiviral production

A second-generation lentiviral expression vector was used to generate second generation CARs (FITC-L-BBz CAR) or EGFP-encoding vectors as control. The FITC-specific CAR construct comprised a human anti-FITC scFv (clone E2), an IgG4 hinge-CH2 (L235D, N297Q)-CH3 spacer fused to a CD28-transmembrane domain and 4-1BB/CD3ζ-endodomain, all linked to a downstream T2A ribosomal skip element and truncated EGF receptor (EGFRt). CAR construct was kindly provided by Dr Michael Jensen from Seattle Children’s Research Institute.

Lentivirus supernatants were produced in 293T cells cotransfected with Pax plasmid (5µg) and envelope VSV-G plasmid (5µg) and CAR or EGFP transfer plasmid using jetPEI® polyethylenimine (Polyplus-transfection®) in 100mm plates (3x10^6^ cells/plate). Viral supernatants were collected and filtered through a 0.45 μm polyethersulfone membrane (Pall laboratory) 48 hours post-transfection.

Supernatants prepared for CAR-T cells assays were concentrated by using Lenti-X™ Concentrator (Takara) and stored at -80°C. Concentrated supernatants were titrated in 293T cells. 293T cells were transduced with the supernatants diluted with DMEM medium at serial dilutions from 10^-2^ to 10^-7^. Percentage of cell transduction (%EGFRt) and viability of cells were studied by flow cytometry.

### 2.6 Transduction of lymphocytes

Activation of T cells was performed using Dynabeads® Human T-Activator CD3/CD28 (Gibco) at bead-to-cell ratio of 1:2. After 48h, transduction was performed with concentrated lentivirus in media containing Polybrene (MerckMillipore), at MOI of 10. Spinoculation was performed using centrifugation at 2,000 rpm for 1 hour as described before (35). The condition-optimizing assays were performed with the EGFP-encoding vectors with the aim of selecting the best condition for transduction. For CAR-T cell production assays, T cells were isolated with the Pan T cell isolation kit (Miltenyi Biotec). To increase the efficacy of transduction and activation stimulus with anti-CD3/CD28, Dynabeads were added at day 0 when the culture started. EGFP or EGFRt expression was analyzed by flow cytometry at day 3 post-transduction.

### 2.7 Vector copy number

DNA from transduced and non-transduced T cells was extracted by using NZY Tissue gDNA isolation kit (Nzytech). Then, Vector Copy Number (VCN) was analyzed by droplet digital PCR (ddPCR) detecting copies of psi (Ψ) signal (FW: 5’CAGGACTCGGCTTGCTGAAG3’, RV: 5’TCCCCCGCTTAATACTGACG3’) normalized to a reference gene as albumin (FW: 5’GCTGTCATCTCTTGTGGGCTG3’ and RV: 5’ACTCATGGGAGCTGCTGGTTC3’), as previously described (36). The reaction was set up in 25μl sample volume containing ddPCR Supermix for Probes (No dUTP, BioRadTM), primers at 900nM final concentration, probes with non-fluorescent quencher (ZEN probes) at a final concentration of 250nM, 2µl of DNA (20-100ng) and nuclease-free water to adjust the sample to the final volume. FAM-labelled probe detecting psi signal:/56-FAM/CG CAC GGC A/ZEN/A GAG GCG AGG/3IABkFQ/(Integrated DNA Technologies) and HEX-labelled probe detecting albumin:/5HEX/CC TGT CAT G/ZEN/C CCA CAC AAA TCT CTC C/3IABkFQ/(Integrated DNA Technologies). Droplets were generated in a QX200 droplet generator (BioRadTM) according to manufacturers’ instructions, obtaining a final volume of 40μl. Finally, droplets were analyzed in a QX200 droplet reader (BioRadTM), and data were processed with QuantaSoft software (BioRadTM). Number of copies/ng of DNA was calculated as previously described (37): Psi VCN x number of total droplets/Albumin VCN.

### 2.8 Functional characterization of CAR-T cells

To study antitumor activity of anti-FITC PB/45RA/CB-derived CAR-T cells *in vitro*, luciferase-expressing NB cells were labeled with 0.2µg per million cells of FITC-conjugated Dinutuximab (anti-GD2) antibody for 30 minutes and then seeded on a 96-well plate with CAR-T cells. CAR-T cells were utilized fresh or cryopreserved and then thawed when used in co-culture. In some assays, CAR-T cells were sorted by anti-EGFRt antibody and magnetic columns (Miltenyi Biotec) to obtain a purity of 90-100% of CAR+ products.

Co-culture experiments were initially carried out with GD2+ tumor cell targets on a white 96-well flat-bottomed plate and 24h later, T cells were added at T cell:tumor cell (effector:target) ratios 2:1, 1:1, 0.5:1, 0.25:1, 0.125:1 without any exogenous cytokines. We employed a luminescence-based method to study the functionality of CAR-T cell, in which the expression of luciferase in NB cell lines acts as a marker of target cell viability as described before (38). Accordingly, 10μg/mL Beetle luciferin (potassium salt, Promega) was added to co-cultured cells and the plate was shaken gently for 10 min in the dark before reading luminescence on a plate reader (Infinite 200 PRO Tecan, Life Sciences). The percentage of surviving tumor cells was calculated as 100×(signal from the sample well—background signal)/(signal from the well containing tumor cells alone—background signal). Luminescence was measured 24h, 48h, 72h and 96h post-coculture.

In addition, after 48h of co-culture of 90% CAR-T+ products with NB cells at ratio 2:1, supernatants were collected to measure the release of cytokines (IL-2, IL-6, IL-10, IFN-γ, TNF-α) by using LEGENDplex™ human Th1 cytokine panel (BioLegend, San Diego, CA). Cell viability was analyzed by flow cytometry.

### 2.9 Genomic stability of CAR-T cells

To evaluate possible aneuploidies, structural variants (SV) and copy number variations (CNV) of CAR-T cell products we employed optical genome mapping (OGM, Bionano Genomics) technology. 2x10^6^ cells were frozen as dry pellet with DNA stabilizer at the beginning (T cells at day 0) and at the end of the protocol (after a 8-day culture). Ultra-high molecular weight DNA was extracted according to SP-G2 bone marrow aspirate DNA Isolation Kit protocol # 90151 and labeled with DLE-1 enzyme according to Bionano Prep Direct Label and Stain-G2 (DLS-G2) Protocol #80046. The quantification of DNA was carried out using a Qubit dsDNA Assay BR or HS Kit (ThermoFisher, #Q32853; Q32854) with small modifications by a Qubit 4.0 Fluorometer (Invitrogen). The DNA was loaded into Saphyr Chip G3.3 #20440 and 4,000-5,000 Gpb was scanned by the Saphyr platform. Molecule quality data were represented by MQR files (**Supplementary Table S1**). Then, the Rare Variant Pipeline (RVP) and Variant Calling were performed on Bionano Solve software (v3.7). Visualization and reporting of SV and CNV were done by Bionano Access (v1.7), using Hg38 as reference genome. The following filters were used: 1) SV filters: overlap precision 3kpb, non-masked SV only, Variant Allele Frequency (VAF) between 0-1, SV found in at least 5 molecules, 0% of SV found in control database, SV found in self-molecules and recommended confidence scores; 2) CNV filters: overlap precision 15kpb, all variants found, recommended confidence scores, min size 500,000 bp and non-masked CNV only. 3) Aneuploidy filters: all and recommended confidence scores.

For the similarity-based study of the SVs, aneuploidies and CNV, a script designed by the bioinformatics unit based on R (Version v4.3.2) and bedtools (version v2.26.0) was used. The ClassifyCNV tool was used to calculate the pathogenicity score of each of the variants found according to American College of Medical Genetics (ACMG) standards (39).

### 2.10 Statistical analysis

Statistical analysis was performed with Prism 8 (GraphPad software). All condition cultures were performed in triplicates. Data are shown as the mean and standard error of mean (SEM)Two-way ANOVA with Tukey’s multiple comparisons test and Bonferroni correction were used for comparison of 3 or more groups. Two-way ANOVA with Sidak’s multiple comparisons test was used for the comparison of 2 groups.

## 3 Results

### 3.1 Impact of culture conditions on proliferation, exhaustion and functional capacity of T lymphocytes from different cell sources.

We aimed to optimize conditions for ex vivo manipulation of human naïve T lymphocytes for using in clinical gene transfer protocols. We compared two sources of clinically available naïve T cells (CB and 45RA enriched peripheral blood leukapheresis products) and cultured them under two sets of cytokine conditions (the classically used IL-2 versus the combination of IL-7+IL-15+IL-21) with or without CD3/CD28 stimulation. PB, a current source of T cells for CAR-T cell manufacturing, was used as control.

First, in preliminary assays, we evaluated the effect of different cytokine conditions such as IL-2, IL-7, IL-7+IL-15 and IL-7+IL+15+IL-21 (**Supplementary Figure S2**) and CD3/CD28 stimulation (**Supplementary Figure S3**) on cytokine-induced T cell proliferation. The IL-7+IL-15+IL-21 condition showed a tendency to favor T-cell proliferation. (**Supplementary Figure S2**). Moreover, significant higher T cell expansion was obtained with CD3/CD28 stimulation in the presence of any cytokine condition (**Supplementary Figure S3**). For this reason, we decided to perform the assays described below comparing the classical cytokine (IL-2) commonly used for ex vivo culture of lymphocytes and the new condition (IL-7+IL-15+IL-21) proposed here, as we mentioned before.

**Figure 1:**
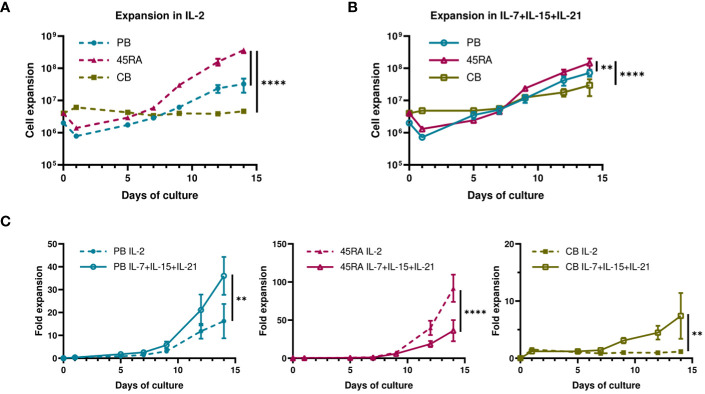
Proliferation of T cells derived from PB, 45RA and CB. **(A)** Total cell expansion (in Log10 scale) of PB, 45RA and CB T cells during 14-day *in vitro* culture with IL-2. **(B)** Total cell expansion (in Log10 scale) of PB, 45RA and CB T cells during 14-day *in vitro* culture with the combination of cytokines IL-7, IL-15 and IL-21. **(C)** Fold expansion (in linear scale) of T lymphocytes derived from the 3 sources in culture with IL-2 *versus* IL7+IL-15+IL-21. Number of replicates: 3. **p value < 0.005. ****p value < 0.0001. CB, cord blood; PB, peripheral blood; 45RA, CD45RA+ fraction post-apheresis.

**Figure 2:**
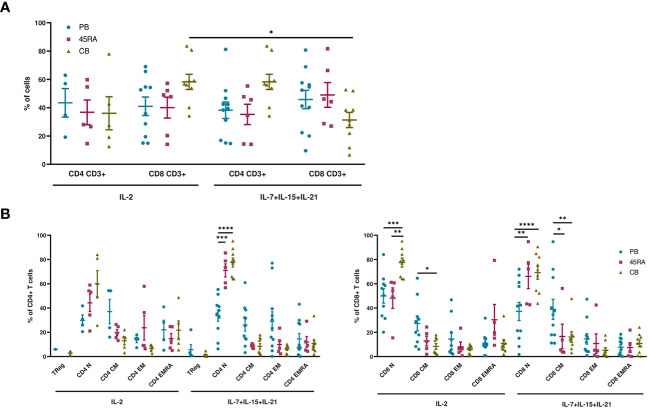
Phenotype of T cells from PB, 45RA and CB after 14-day culture. **(A)** Phenotype of T cells (CD4+:CD8+ ratio) after a 14-day culture with IL-2 or IL-7+IL-15+IL-21. **(B)** T cell subsets of CD4+ and CD8+ lymphocytes after a 14-day culture with IL-2 vs. IL-7+IL-15+IL-21. Number of replicates: 11(PB), 6(45RA), 9(CB). *p value < 0.05. **p value < 0.005. ***p value < 0.0005. ****p value < 0.0001. CB, cord blood; PB, peripheral blood; 45RA, CD45RA+ fraction post-apheresis.

When cultured with IL-2 or IL-7+IL-15+IL-21 (**Figure 1**), 45RA T cells showed higher expansion than CB T cells (**Figures 1A, B**), independently of the cytokine conditions (**Figure 1C**). A trend toward increased T-cell proliferation was observed with the 3-cytokine combination for all cell sources (**Figure 1C**). We found some variability related to the cell donor (**Supplementary Figure S4**). Next, we studied the proportions of different T cell subsets at the end of the culture period compared to the phenotype at day 0 of culture. At day 0, the T cell phenotype was not totally divided into CD4+ and CD8+ cells since we observed an important CD4-CD8- population depending on the source of cells (23.9% from PB, 44.1% from 45RA and 41.8% from CB) (**Supplementary Figure S5A**). Naïve cells tended to be more abundant in T cells obtained from different donors of 45RA fraction (n=7) (59.3% in CD4+ and 66.4% in CD8+ cells) and CB (n=9) (66% in CD4+ and 29.3% in CD8+ cells) than in T cells from PB (n=5) (41.5% in CD4+ and 28.1% in CD8+ cells) at day 0 (**Supplementary Figure S5B**). The proportions of CD4+ or CD8+ cells were not significantly influenced by the presence of the cytokines (**Figure 2A**). Regarding immune phenotype markers of maturation, we studied in prior assays how different combinations of cytokines affected maturation, obtaining promising results in which cultures with IL-7+IL-15+IL-21 maintained a naïve phenotype of T cells better than other conditions (**Supplementary Figure S6**). When analyzing this cytokine condition against the commonly used IL-2, PB T cells showed increased memory phenotypes, with the lowest percentages of naïve T cells (CD4+: 29.8% with IL-2 vs 33.7% with IL-7+IL-15+IL-21; CD8+: 49.9% with IL-2 vs 33.7% with IL-7+IL-15+IL-21) (**Figure 2B**). On the contrary, 45RA-derived and CB-derived T cells produced higher percentages of naïve T cells: 44.2% with IL-2 vs. 71% with IL-7+IL-15+IL-21 in CD4 + 45RA T cells, and 60% with IL-2 vs. 77.5% with IL-7+IL-15+IL-21 in CD4+ CB T cells. Similar percentages were seen for CD8+ T cells. The differences in naïve T cells among CD4+ T cells were statistically significant only under IL-7+IL-15+IL-21 conditions (**Figure 2B**). We did not find significant differences in T regulatory cell levels comparing cell sources and cytokine conditions (**Figure 2B**).

**Figure 3:**
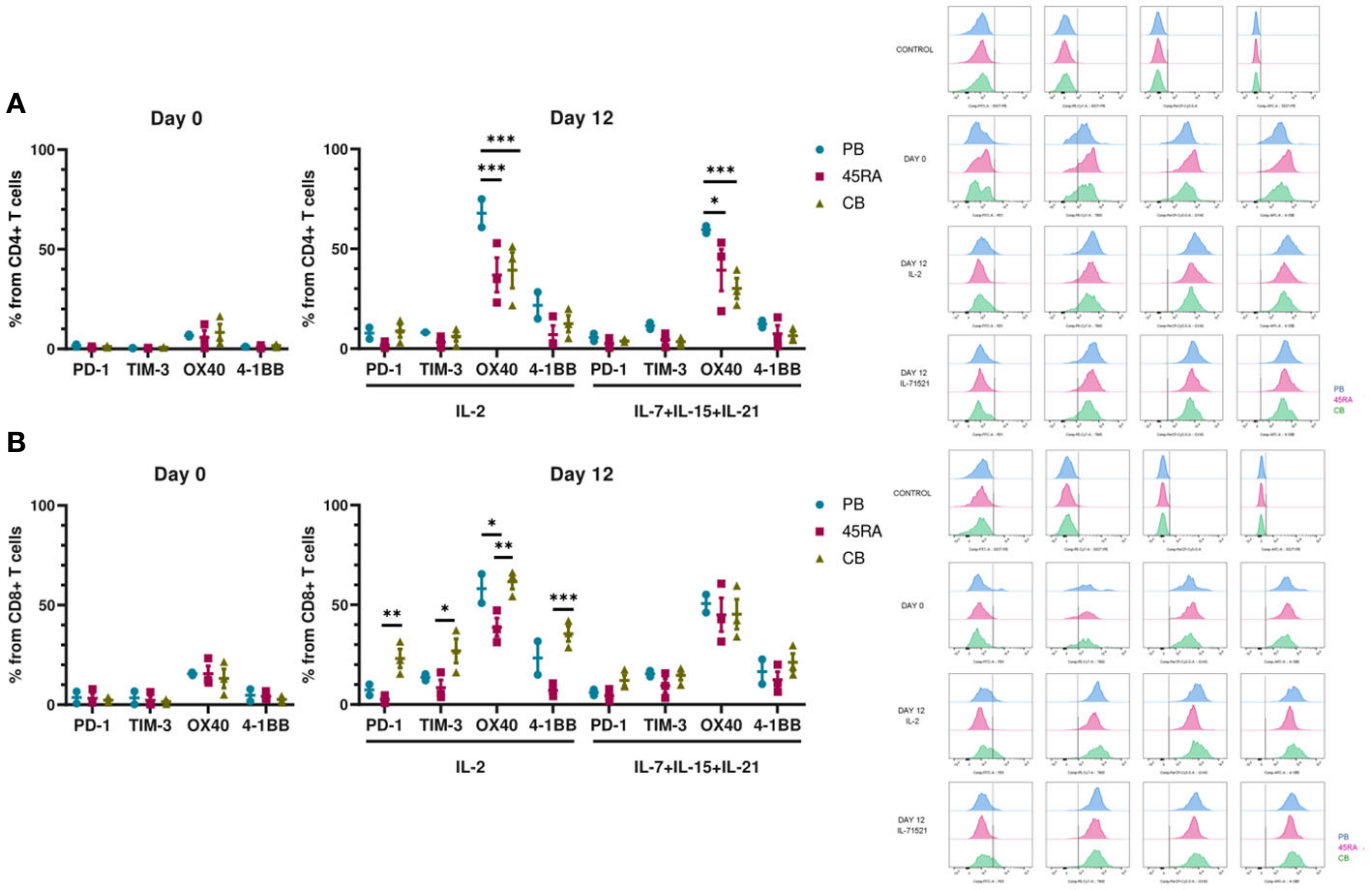
Activation and exhaustion of T cells after 12 days of culture *in vitro* with IL-2 vs. IL-7+IL-15+IL-21. **(A)** Activation and exhaustion markers of CD4+ T cells at day 0 and day 12 of culture with IL-2 vs. IL-7+IL-15+IL-21. **(B)** Activation and exhaustion markers of CD8+ T cells at day 0 and day 12 of culture with IL-2 vs. IL-7+IL-15+IL-21. Cytometry histograms from days 0 and 12 along with isotype controls are shown on the right of the figure. Number of replicates: 3. *p value < 0.05. **p value < 0.005. ***p value < 0.0005. CB, cord blood; PB, peripheral blood; 45RA, CD45RA+ fraction post-apheresis.

We were also interested in the expression of immune phenotype markers of T-cell activation and exhaustion after 14 days in culture (**Figure 3**). We analyzed the pattern of co-inhibitory (PD-1 and TIM-3) and co-stimulatory (OX40 and 4-1BB) receptors. Low proportions of CD4+ T cells seemed to express PD-1 and TIM-3 independently of cell source or cytokine condition (**Figure 3A**). On the other hand, higher percentages of CD8+ CB cells expressed the similar levels of co-inhibitory receptors when cultured with IL-2 compared to those cultured with IL-7+IL-15+IL-21 (22.3% PD-1 and 27.3% TIM-3, versus 9.1% of PD-1 and 16.1% of TIM-3, respectively) (**Figure 3B**). The proportions of CD4 or CD8 T cells expressing the co-stimulatory markers OX40 or 4-1BB were comparable among the different cell sources and cytokine conditions.

**Figure 4:**
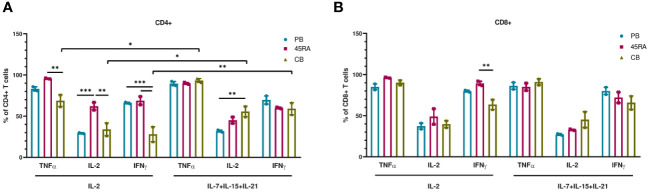
Intracellular cytokine expression in T cells after *in vitro* culture with IL-2 vs. IL-7+IL-15+IL-21 during 14 days. **(A)** Intracellular cytokine expression in CD4+ T cells. **(B)** Intracellular cytokine expression in CD8+ T cells. Number of replicates: 3. *p value < 0.05. **p value < 0.005. ***p value < 0.0005. CB, cord blood; PB, peripheral blood; 45RA, CD45RA+ fraction post-apheresis.

We finally used the cells obtained at the end of the culture period to functionally test their capacity to produce cytokines in response to mitogens (**Figure 4**). 45RA-derived CD4 cells cultured with IL-2 (but not with the 3-cytokine cocktail) expressed higher levels of either TNF-α, IL-2 or IFN-γ compared to CB or PB. Only in CB cultures, the combination of IL-7+IL-15+IL-21 resulted in CD4 T cells capable of producing higher levels of TNF-α, IL-2 or IFN-γ compared to that of cultures with only IL-2 (**Figure 4A**). We found no significant differences in the proportion of CD8 T lymphocytes expressing intracellular cytokines when comparing cell source and cytokine conditions, except for IFN-γ, which showed the highest expression levels in cultures initiated with 45RA T cells from leukapheresis and stimulated with IL-2 (**Figure 4B**).

The conclusion of this set of experiments was that the best conditions for ex vivo manipulation of human naïve T lymphocytes in clinical gene transfer protocols required CD3/CD28 stimulation. The presence of cytokines, either only IL-2 or the cocktail of three cytokines affected the resulting T cells both immunophenotypically and functionally. Moreover, after analyzing all the results obtained as a whole, taking into account all the studied parameters and the two cytokine conditions, we concluded that the best cytokine condition was IL-7+IL-15+IL-21, since our priority was not obtaining a massive cell expansion but a more naïve T cell phenotype.

### 3.2 Gene transfer into ex vivo cultured T cells

**Figure 5:**
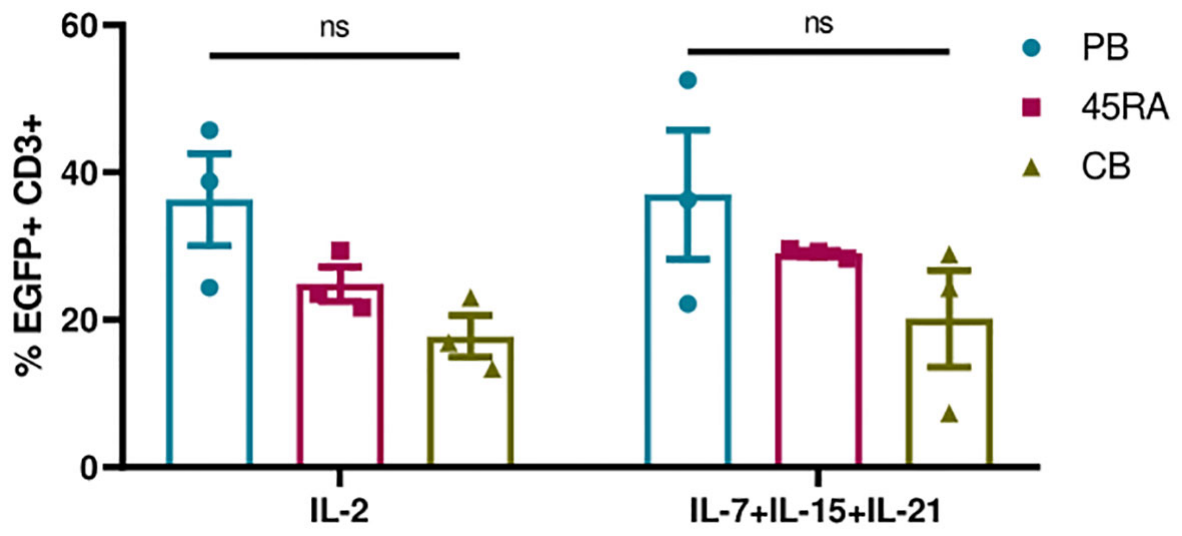
Transduction efficiency of EGFP LV in T cells after 5 days in culture with IL-2 vs. IL-7+IL-15+IL-21 and anti-CD3/CD28 stimulation. Number of replicates: 3. ns, not significant; CB, cord blood; PB, peripheral blood; 45RA, CD45RA+ fraction post-apheresis.

Next, we transduced T lymphocytes from PB, 45RA fraction and CB, with an EGFP-expressing lentiviral vector (LV) under the above-mentioned culture conditions. First, we analyzed the importance of adding cytokines and CD3/CD28 stimulation to obtain higher percentages of transduction (**Supplementary Figure S7**). EGFP expression was followed from day 5 after transduction. We were able to appreciate a tendency in the transduction capacity of the different T lymphocyte donor sources, following the same pattern with both cytokine conditions. A higher transduction capacity could be observed in PB-derived T cells, and the lowest in CB-derived T cells (**Figure 5**). Regarding culture conditions, the combination of IL-7+IL-15+IL-21 promoted slightly higher percentages of transduction than IL-2 (37% in PB T cells, 29% in 45RA T cells and 20% in CB T cells versus 36.3%, 24.9% and 17.7%, respectively). In none of the cases the differences observed were significant (**Figure 5**).

### 3.3 Generation of CAR-T cells from two clinical sources and comparison according to safety immunophenotype, and functional capacities

**Figure 6:**
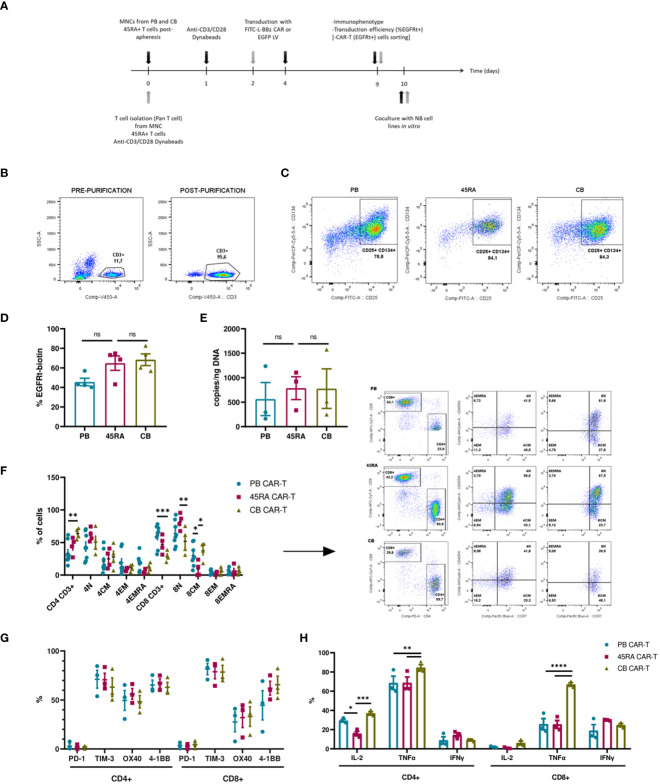
CAR-T cell production. **(A)** Experimental design. Black arrows show the steps followed when production starts with mononuclear cells (MNC) (fresh or thawed cells). Grey arrows show the steps followed when the production starts with T cells (after purification by Pan T cell kit) (fresh or thawed samples). **(B)** CD3+ cell percentage before and after T cell purification by Pan T cell kit. **(C)** Activation of T cells after 2-day culture with anti-CD3/CD28 Dynabeads showing the expression of CD25 and CD134 markers. **(D)** Transduction efficiency of FITC-L-BBz CAR LV in T cells assessed by flow cytometry. Number of replicates: 4. **(E)** VCN analysis by ddPCR of T cells transduced with FITC-L-BBz CAR LV: copies/ng of DNA in CAR-T cell products. Number of replicates: 3. **(F)** Phenotype of CAR-T (CD4+ and CD8+) cells. Dot-plots representing the T-cell subpopulations of the different products. Number of replicates: 8 (PB), 4 (45RA), 4 (CB). **(G)** Exhaustion status of CAR-T cells prior co-cultures. Number of replicates: 3. **(H)** Intracellular cytokine expression in CAR-T cells after proliferation, activation and transduction *in vitro*. Number of replicates: 3. *p value < 0.05. **p value < 0.005. ***p value < 0.0005. ****p value < 0.0001. ns: not significant. CB, cord blood; PB, peripheral blood; 45RA, CD45RA+ fraction post-apheresis; T_N_, naïve T cell; T_CM_, central memory T cells; T_EM_, effector memory T cells; T_EMRA_, terminal effector T cells.

We developed a protocol to obtain anti-FITC CAR-T cells from PB, 45RA and CB samples (**Figure 6A**) based on the above-mentioned conditions. To improve T cell transduction, CD3+ cells were purified from the different sources up to 90% purity (**Figure 6B**). After 2-3 days of co-stimulation with Dynabeads and IL-7+IL-15+IL-21, around 80% of T cells from all sources expressed early activation markers (CD134+ CD25+) (**Figure 6C**), which allowed subsequent transduction with an FITC-L-BBz CAR LV. Transduction efficiency showed a higher trend for 45RA (64.9%) and CB (68.4%) than for PB (45.7%) T cells (**Figure 6D**).

As part of a deep characterization of the CAR-T products in terms of safety, Vector copy number was analyzed by ddPCR, obtaining results that correlated with the transduction efficiency assessed by flow cytometry, with 787copies/ng (45RA), 777 copies/ng (CB) and 564copies/ng of DNA (PB) (**Figure 6E**). Charts and data from the analysis of the results of ddPCR with the controls of non-transduced T cells are shown in **Supplementary Figure S8**.

We also did an ample immunophenotypic characterization of the CAR-T cell products. First, we analyzed the CD4/CD8 ratio of CAR-T cells obtained from the various sources. PB CAR-T cells showed a 40/60 ratio versus the 50/50 ratio of 45RA CAR-T and 70/30 ratio of CB CAR-T cells. CAR-T cells from all sources presented a naïve phenotype, being 45RA CAR-T cells the product with the highest levels of naïve T cells (61.6% in CD4+ and 82.1% in CD8+ cells) (**Figure 6F**) (a control sample for this analysis is shown in **Supplementary Figure S9**). Moreover, we analyzed exhaustion markers of CAR-T cells after 8 days of culture and prior to co-culture assays (dot plots from this analysis are shown in **Supplementary Figure S10**). In general, CAR-T cells from the three sources presented similar expression levels, CD4+ CAR-T showed no significantly higher levels of co-stimulatory receptors than CD8+ CAR-T. PD-1 levels were low, while TIM-3 was high in CD4+ and CD8+ CAR-T cells (**Figure 6G**). CAR-T cells were also studied to test their capacity to produce cytokines when activated with an unspecific stimulus (leukocyte activation cocktail). CB CAR-T cells expressed higher levels of IL-2 and TNF-α than PB and 45RA CAR-T cells. 45RA CAR-T showed higher levels of IFN-γ than PB or CB CAR-T (**Figure 6H**), although the difference was not statistically significant.

**Figure 7:**
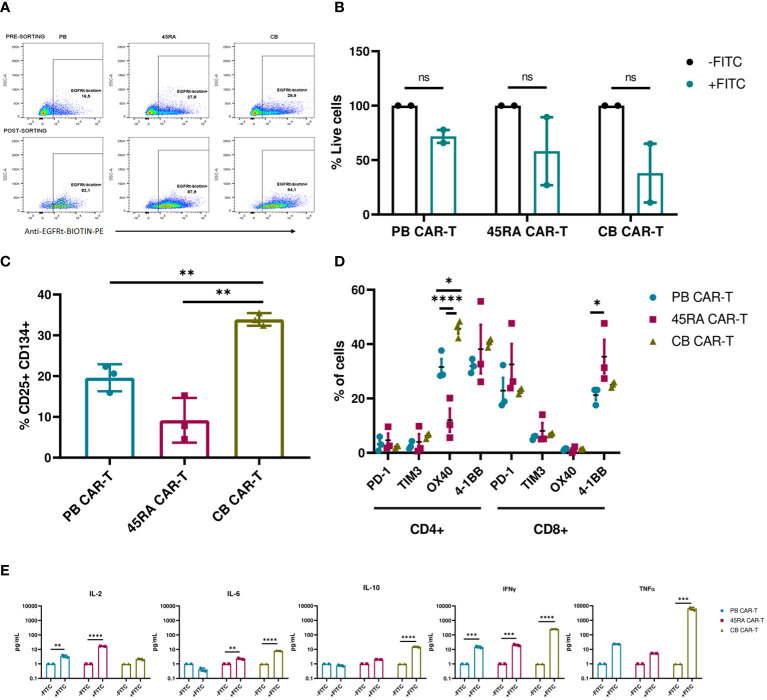
Functionality of enriched CAR-T cells (90% CAR-T) derived from PB, 45RA+ cells and CB. **(A)** Sorting of CAR-T cells by selecting EGFRt+ cells. **(B)** Luciferase-expressing LAN-1 cell viability by flow cytometry in co-culture with CAR-T cells at 2:1 CAR : NB ratio. Data normalized to the control condition of co-culture without Dinutuximab-FITC. **(C)** Activation markers of CAR-T cells in co-culture with NB treated with Dinutuximab-FITC. **(D)** Exhaustion and activation of CD4+ and CD8+ CAR-T cells in co-culture with NB treated with Dinutuximab-FITC. **(E)** Cytokines released by CAR-T cells in the cultures with NB at ratio 2:1 treated or not with Dinutuximab-FITC. Normalized to the control condition without FITC. Number of replicates: 3. *p value < 0.05. **p value < 0.005. ***p value < 0.0005. ****p value < 0.0001. ns, not significant; CB, cord blood; PB, peripheral blood; 45RA, CD45RA+ fraction post-apheresis.

To study the functionality of anti-FITC CAR-T cells, luciferase-expressing LAN-1 neuroblastoma cells were co-cultured with CAR-T cells at a 2:1 ratio in the presence of an anti-GD2-FITC antibody (Dinutuximab). In previous assays, we defined the dose of antibody required to obtain the maximum possible percentage of GD2+ cells with the minimum quantity of antibody (**Supplementary Figure S11**). CAR-T cell products were enriched by sorting EGFRt+ T cells obtaining 90% purity from all the sources (**Figure 7A**). The viability of neuroblastoma cells in the co-cultures dropped when treated with Dinutuximab-FITC. However, the viability differences were not significant compared to the controls without the targeting treatment (**Figure 7B**, **Supplementary Figure S12**). Expression of exhaustion/activation markers by CAR-T cells was studied after 24h in co-culture with NB. In general, CD4+ CAR-T cells expressed high levels of co-stimulatory receptors and low levels of co-inhibitory receptors. Although differences of these receptors between the different products showed a pattern, in most cases the differences were not statistically significant. CD4+ CB CAR-T cells presented the highest proportion of cells expressing co-stimulatory receptors such as OX40 (significantly higher compared to the CAR-T from the other two sources;45.7% vs. 31.6% in PB CAR-T and 12% in 45RA CAR-T) and 4-1BB (40.5% vs. 31.9% in PB CAR-T and 38.1% in 45RA CAR-T). CD8+ CAR-T cells exhibited elevated levels of PD-1 and 4-1BB, while TIM3 and OX40 levels were low. CD8 + 45RA CAR-T expressed the highest levels of PD-1 (32.5% vs. 22.9% in PB CAR-T and 22.7% in CB CAR-T) and 4-1BB (35.4% vs. 21.2% in PB CAR-T and 25% in CB CAR-T) (**Figure 7D**).

CAR-T cells derived from CB were the most activated product according to the expression of early activation markers (33.9% CD25+CD134+ cells vs. 19.6% in PB CAR-T and 9.2% in 45RA CAR-T) (**Figure 7C**) and cytokines released into the supernatants (**Figure 7E**), in line with the highest decrease of viability obtained with CB CAR-T cells (**Figure 7B**). Cytokine levels were normalized to the control co-cultures of each condition without Dinutuximab-FITC. Levels of IL-2, IL-6 and IL-10 were the lowest within all the measured cytokines, with the maximum IL-2 released by 45RA CAR-T (16.7 pg/ml) and the maximum IL-6 (8 pg/ml) and IL-10 (15.35 pg/ml) released by CB CAR-T. IFN-γ levels were high in cultures of CAR-T cells from all sources in the presence of targeting, with significant differences with the controls. The maximum IFN-γ was released by CB CAR-T cells, correlated again with the NB viability results obtained after co-culture. They also showed the highest level of TNF-α (6,545 pg/ml) in culture with NB treated with Dinutuximab-FITC compared to PB CAR-T (23.2 pg/ml) and 45RA CAR-T (5.3 pg/ml) normalized to control without Dinutuximab-FITC (**Figure 7E**). The analyzed parameters related to the targeting of the CAR-T cell towards NB tumor cells resulted in low values, however, they are consistent among them since they correlate with the most functional product based on the observed NB viability. This set of experiments allowed us to determine the differences between the sources.

**Figure 8:**
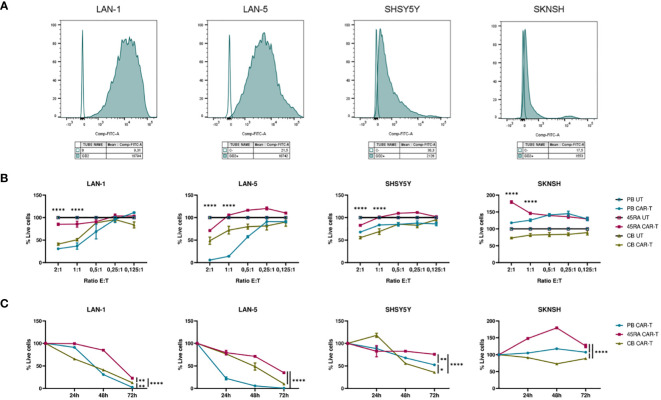
Functionality of CAR-T cells derived from PB, 45RA+ cells and CB normalized with T cells (42% CAR-T cells). **(A)** GD2 antigen density on the surface of different neuroblastoma (NB) cell lines by flow cytometry. **(B)** NB viability after 48h in co-culture at different effector:target ratios. Data normalized to the conditions of co-culture with untransduced T cells. **(C)** NB viability after 24h, 48h and 72h in co-culture at 2:1 effector:target ratio. Number of replicates: 3. *p value < 0.05. **p value < 0.005. ****p value < 0.0001. CB, cord blood; PB, peripheral blood; 45RA, CD45RA+ fraction post-apheresis.

Furthermore, we co-cultured CAR-T products (with a 42% of CAR+ cells after normalizing with untransduced T cells from same donors) at different ratios (2:1, 1:1, 0.5:1, 0.25:1, 0.125:1) with different NB cell lines treated with Dinutuximab-FITC (**Figure 8**). PB and CB CAR-T showed the highest cytolytic effect after 48h of co-culture with NB cells treated with anti-GD2-FITC (**Figure 8B**). The viability obtained in the assays correlated with GD2 antigen density on the membrane of NB cell lines. SKNSH showed the lowest density of GD2 (MFI: 1,553), while LAN-1 (MFI: 18,794) and LAN-5 (MFI: 10,742) cell lines had the highest expression (**Figure 8A**). The lowest viability obtained in NB cell lines was 30% in LAN-1 cells when co-cultured with PB CAR-T and 41% when co-cultured with CB CAR-T; 57% in LAN-5 co-cultured with PB CAR-T and 48% when co-cultured with CB CAR-T; 55% in SHSY5Y cells (MFI: 2,126) when co-cultured with CB CAR-T and 67% when co-cultured with PB CAR-T; and 73% in SKNSH cells when co-cultured with CB CAR-T and 117% (cells were proliferating) when co-cultured with PB CAR-T (**Figure 8B**). Furthermore, focusing on the progress of cell death from day 0 to 72h after co-culture, we observed that, as already seen in **Figure 8B**, the viability of LAN-1 and LAN-5 cell lines at 72h was reduced to 0 when co-cultured with PB CAR-T), whereas it was only reduced to 23% (LAN-1) and 35% (LAN-5) with 45RA CAR-T, and to12% and 10%, respectively, with CB CAR-T. Viability was also reduced in SHSY5Y cell line, although reaching values of 52.5%, 76% and 35.8% at 72h in the presence of PB, 45RA and CB CAR-T products, respectively. However, in the case of SKNSH, no decrease in tumor viability with time after co-culture was observed (**Figure 8C**).

### 3.4 Genome stability of CAR-T cell products

To study the genomic integrity of CAR-T cell products, we performed optical genome mapping (OGM), a novel genomic technology which detects rare structural variations with higher sensitivity and resolution than gold standard techniques. We analyzed PB, 45RA and CB CAR-T products after 8 days of culture, and compared with T cells from donor samples at day 0. In order to reach a VAF up to 0.5%, a range of 4,000 to 5,000 Gpb was scanned per sample. The effective coverage ranged between 1350-1300X, 900-800X and 1300-1000X for PB, 45RA and CB samples, respectively (**Supplementary Table S1**).

Details of aneuploidies, SVs and CNV detected are shown in the **Supplementary Table S2**. Importantly, no aneuploidies were found in the samples studied. Some SVs were present in paired samples of control and transduced cells in PB, 45RA and CB (all of them of uncertain significance or benign) pointing out that they were not a consequence of CAR-T cell manufacturing. SVs found only in the control samples show a clonal selection based on cellular fitness.

**Figure 9:**
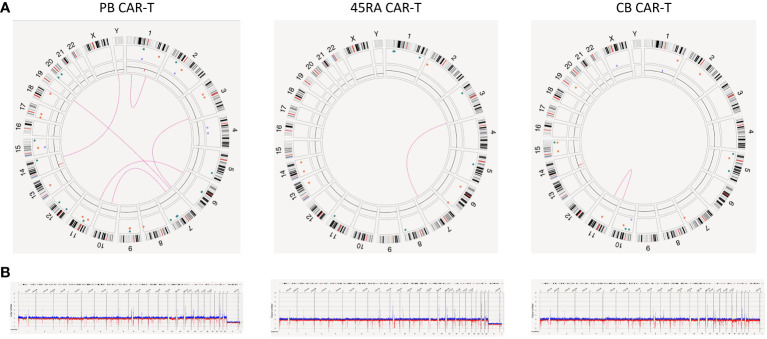
Genome stability of CAR-T products. **(A)** Circus plot showing SVs found in both CAR-T products and control samples derived from PB, 45RA and CB. Green: insertion, orange: deletion, light blue: inversion, purple: duplication, pink: translocation. **(B)** Whole genome showing structural variants found in both CAR-T products and control samples derived from PB, 45RA and CB. Dark blue: CNV gain. Red: CNV loss.

However, the most relevant findings regarding the safety of manufactured cell products was obtained in the study of the new variants found in CAR-T products (**Figure 9**). We found several SV (deletions, insertions, duplications and translocations) in the three types of CAR-T cells. Nevertheless, upon a manual revision of each SV, we concluded that most of them appear to be false positives due to VAF<0.1, low confidence, size lower or close to limit of detection (5 kb for insertions and 7 kb for deletions), few DLE-1 labels or not reliable molecules. As the software did not exclude those that were artifacts, we filtered out some of them to draw definitive conclusions. After filtering out, two translocations were found in PB CAR-T cells [chr7:64,820,257; chr10:57,453,593 (0.19 VAF, 0.21 confidence) and chr7:18,849,498; chr19:23,828,061.5 (0.1 VAF, 0.18 confidence)] affecting genes as ZNF138 and HDAC9; and AC139769, which are involved in transcription regulation and histone deacetylase binding, respectively. Furthermore, a deletion was found [chr17: 29,488,145-29,522,318 (0.39 VAF, 0.99 confidence)] affecting TAOK1, classified as likely pathogenic. This gene is related to transferase and tyrosine kinase activity and involved in mitotic G2 DNA damage checkpoint signaling. In the case of 45RA CAR-T cells, one translocation was found [chr3:195,979,587; chr7:131,665,402 (0.16 VAF, 0.32 confidence)] affecting the pseudogene SDHAP1, and two insertions were detected [chr1: 477,561,129-477,591,173 (0.48 VAF, 0.99 confidence) and chr5: 76,775,024-76,799,943 (0.46 VAF, 0.99 confidence)] affecting non-coding regions. Finally, a deletion was found in CB CAR-T cells [chr15:28,215,386-28,286,638 (0.18 VAF, 0.99 confidence)] affecting HERC2 gene linked to ligase activity and ubiquitin protein ligase binding. Regarding the insertion of the lentiviral vector used to transduce T cells, we were unable to track it by OGM as its size is below the detection limit of the technology.

## 4 Discussion

Adoptive cell transfer with CAR-T cells has shown outstanding achievements for treating hematological malignancies with the approval of anti-CD19 CAR therapies by the FDA. However, the use of CARs to target adult or pediatric solid tumors has shown less efficacy due to the lack of specific antigens and the suppressive tumor microenvironment (6), which can lead to low persistence and efficacy of CAR-T cells.

In this work, we developed different approaches to confront these disadvantages of CAR-T cells for the treatment of solid tumors. First, we optimized culture conditions to obtain more stem-like T cell subsets, which persist in the host and show functionality due to their self-renewal capacity and their multipotency (21–23). Other authors have presented different strategies, such as inhibition of PI3K–mTOR–AKT, BTK or tyrosine kinase signaling, shorter culture duration, or special culture conditions to optimize CAR-T cell phenotype (40). Here, we show how the combination of cytokines IL-7+IL-15+IL-21 favors the naïve phenotype of T cells from the three donor sources employed. Other authors have described the use of IL-7 and IL-15 to maintain a more naïve phenotype and proliferation in these cells (41, 42), and others also added IL-21 to this cytokine combination to help block the differentiation of naïve T cells (27, 43–47). Del Bufalo et al. (37) employed IL-7+IL-15 in the culture of anti-GD2 CAR-T cells, showing predominant central memory and effector memory phenotypes. Therefore, we highlight the importance of adding IL-21 to the culture to obtain a more naïve phenotype. This combination of cytokines plus anti-CD3/CD28 stimulation promoted a higher proliferation of T cells than that obtained with the commonly used IL-2, as other groups have previously reported for IL-7+IL-15 (22, 24, 29, 48) or also for IL-21 (34, 49, 50).

Second, this study analyzed two potential cell sources of T cells with a more naïve phenotype, cord blood and 45RA fraction post-apheresis from healthy donors. Both sources had higher percentages of naïve T cells than PB T cells. Several groups have produced data regarding the efficacy of CD19-specific CB CAR-T cells, proving the cytolytic effect of CB-derived and -modified T cells both *in vitro* and *in vivo* in models of B-lineage acute lymphoblastic leukemia (B-ALL) (42, 51). In agreement, our results suggest that 45RA and CB T cells express lower values of co-stimulatory and co-inhibitory receptors than PB T cells, which could sustain their persistence in the host. Moreover, CB CAR-T cells present high levels of OX40 and 4-1BB co-stimulatory receptors, showing their capacity to be activated and functional. This finding is in accordance with other authors who claimed that CD8+ CB T cells generally showed a higher proliferative and cytotoxic potential than CD8+ PB T cells (34).

Third, we proposed the monoclonal antibody Dinutuximab conjugated with FITC as a specific strategy to facilitate NB targeting through recognition by anti-FITC CAR-T cells. However, other approaches based on MABG (an analogue of MIBG) developed before (52–54) could also be used to target NB in combination with FITC and anti-FITC CAR-T cells. Some authors have developed anti-GD2 CAR-T cells with limited efficacy (37, 55). Del Bufalo et al. (37) reported that 33% of the patients had a complete response, 30% had a partial response,19% had stable disease, and 19% had no response. They also highlighted that patients who relapsed did not lose GD2 expression, suggesting that CAR-T cell treatment failure in NB is due to resistance, unlike in leukemia, where is mainly caused by antigen loss. To approach this issue, in this work we have developed CAR-T products with a more naive phenotype, which we hypothesize will improve host persistence. Furthermore, the advantage that these anti-FITC CARs can provide is the possibility of employing different targeting elements at the same time against different proteins expressed on the tumor cell membrane. Accordingly, different monoclonal antibodies conjugated to FITC targeting different tumor antigens could be combined to promote a polyclonal CAR-T response with greater cytolytic activity.

Forth, we analyzed the genome stability of CAR-T cell products to prove the safety of ex vivo culture and transduction of T cells. Taking into account the FDA announcement (56) concerning the risks of T-cell malignancies after treatment with autologous CAR-T targeting BCMA or CD19, it will be important to incorporate robust methods to detected VCN and structural variants in the CAR-T cell production workflows. Using the OGM technology, we were able to examine aneuploidies, SVs and CNV events in the CAR-T cell products, even when they appeared in a low percentage of clones. After filtering, we were confident of capturing some unexpected SVs. In summary, within the limits and confidence of OGM technology, our unbiased analysis of the overall genomic structure of CAR-T cell products revealed a single event with potential pathogenic implications only in one of the analyzed donor sources (PB). However, the other SVs found with uncertain significance, although unexpected to be transforming events, should be taken into account for the manufacture of cell therapy products. Importantly, some of the SVs found in CART cells were present before the culture, pointing to the need to validate the chromosomal stability of donor sources before manufacturing cell therapy products. Although OGM has provided a very comprehensive landscape of genome structure in CAR-T cells, further analyses will be necessary to assess safety and risk of genotoxicity in long-term clinical outcomes. Recently, Canarutto et al. (57) also validated the sensitivity of Optical Genome Mapping to analyze genome integrity after CD4+ T cell gene editing. The results of this work showed that PB and CB CAR-T exhibited higher cytotoxicity than 45RA CAR-T cells. Additionally, CB CAR-T cells released higher levels of cytokines in practically all conditions. Specifically, CB CAR-T cells released the highest amount of TNF-α and IFN-γ. This result is accordance with a report showing that CB T cells secreted more IFN-γ and decreased levels of IL-2 (42). On the contrary, 45RA CAR-T cells were the least activated product (CD25+ CD134+ markers) and showed higher levels of PD1 than PB or CB CAR-T. Taken altogether, these results suggest that 45RA CAR-Ts are, in general, more exhausted than CAR-T cells from other sources, with less cytolytic activity, lower levels of secreted TNF-α and IFN-γ, and higher levels of IL-2 than PB/CB CAR-T cells. These characteristics may be due to their naïve phenotype (a higher proportion of naïve cells than in the other products) as previously reported by other authors (58).

Finally, taking into consideration all the results presented here, we recommend the use CB T cells to produce allogenic CAR-T cell products. Allogenic CAR-T cells offer advantages in treatment timelines and costs (59). Universal CAR-T cells could trigger graft versus host disease associated with the disparity of TCR on the surface of T cells between donor and recipient (60). To avoid this adverse event, CRISPR/Cas9 technology can be used to knock out the endogenous TCR of T cells (61–63). This approach is based on electroporation of activated T cells, which is known to cause high levels of toxicity that could impair the expansion protocols of these cell products. Therefore, following the findings published by Nahmad et al. (64), further studies are required to continue this work including the electroporation of T cells in the anti-FITC CAR-T production protocol in order to study the effect of this technique to the final product.

## Data availability statement

The original contributions presented in the study are included in the article/**Supplementary Material**. Further inquiries can be directed to the corresponding authors.

## Ethics statement

The studies involving humans were approved by Comité de Ética de la Investigación con medicamentos del Hospital Infantil Universitario Niño Jesús de Madrid. The studies were conducted in accordance with the local legislation and institutional requirements. The human samples used in this study were acquired from public Blood bank: Centro de Transfusión de la Comunidad de Madrid, Banco de Sangre del Hospital Niño Jesús. Written informed consent for participation was not required from the participants or the participants’ legal guardians/next of kin in accordance with the national legislation and institutional requirements.

## Author contributions

LG-G: Data curation, Formal analysis, Investigation, Methodology, Software, Validation, Visualization, Writing – original draft, Writing – review & editing. ES: Formal analysis, Validation, Writing – review & editing, Investigation, Methodology, Software, Visualization. MI: Methodology, Writing – review & editing, Investigation, Software. KP: Methodology, Writing – review & editing, Investigation. CA-S: Methodology, Writing – review & editing, Investigation. JG-M: Formal analysis, Software, Validation, Writing – review & editing. BM-A: Investigation, Methodology, Supervision, Writing – review & editing. MR: Conceptualization, Funding acquisition, Project administration, Resources, Supervision, Validation, Writing – review & editing, Formal analysis, Investigation, Methodology. ÁG-M: Conceptualization, Investigation, Project administration, Supervision, Validation, Writing – review & editing, Formal Analysis, Methodology.
