# Supplementary material for: Choosing T-cell sources determines CAR-T cell activity in neuroblastoma
Source: Front Immunol. 2024 Mar 27;15:1375833. doi: 10.3389/fimmu.2024.1375833 (PMC11004344; doi:10.3389/fimmu.2024.1375833)
Supplement: Supplementary file 1 [file DataSheet_1.pdf]

Supplementary Material

1 Supplementary Figures and Tables

1.1 Supplementary Figures

A

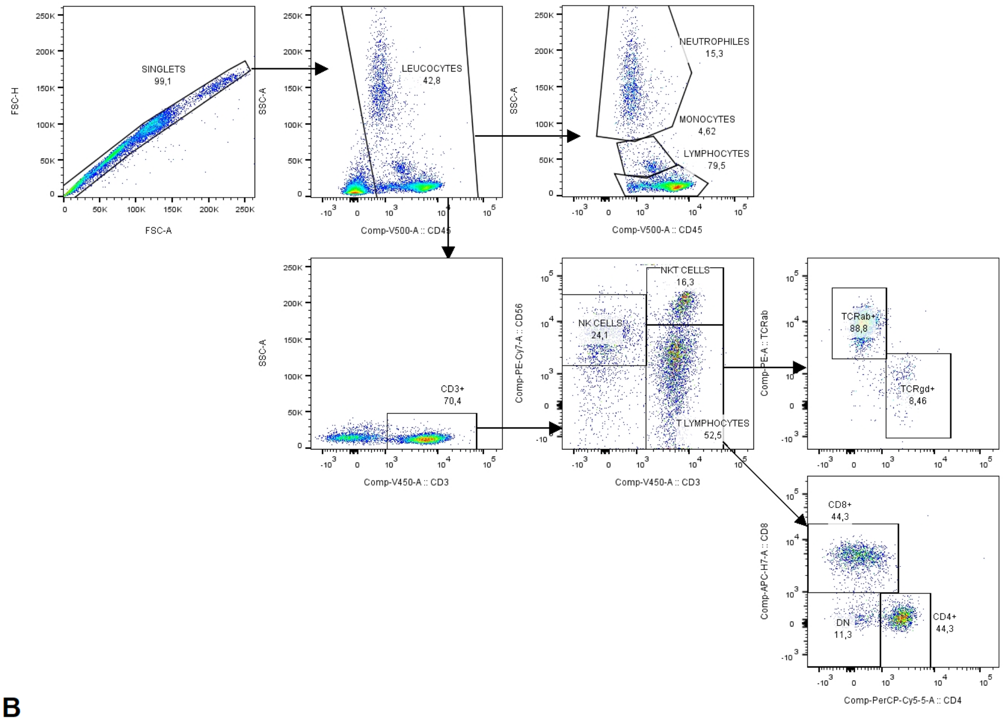
SINGLETS
99,1
FSC-H
FSC-A
LEUCOCYTES
42,8
SSC-A
Comp-V500-A :: CD45
NEUTROPHILES
15,3
MONOCYTES
4,62
LYMPHOCYTES
79,5
CD3+
70,4
Comp-V450-A :: CD3
NKT CELLS
16,3
NK CELLS
24,1
T LYMPHOCYTES
52,5
Comp-PE-Cy7-A :: CD56
TCRab+
88,8
TCRgd+
8,46
Comp-PE-A :: TCRab
CD8+
44,3
DN
11,3
CD4+
44,3
Comp-APC-H7-A :: CD8
Comp-PerCP-Cy5-5-A :: CD4

B

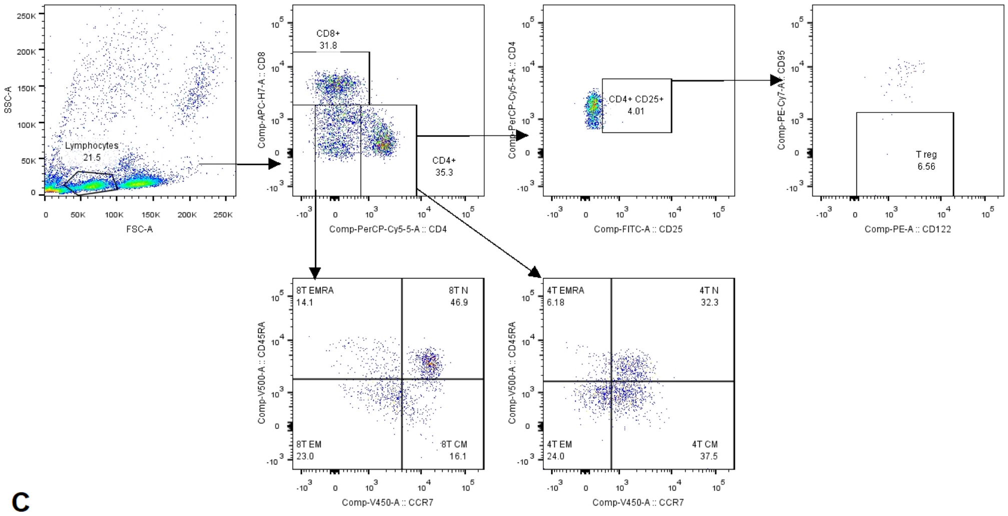
Lymphocytes
21.5
SSC-A
FSC-A
CD8+
31.8
CD4+
35.3
Comp-APC-H7-A :: CD8
Comp-PerCP-Cy5-5-A :: CD4
CD4+ CD25+
4.01
Comp-FITC-A :: CD25
T reg
6.56
Comp-PE-Cy7-A :: CD95
Comp-PE-A :: CD122
8T EMRA
14.1
8T N
46.9
8T EM
23.0
8T CM
16.1
4T EMRA
6.18
4T N
32.3
4T EM
24.0
4T CM
37.5
Comp-V500-A :: CD45RA
Comp-V450-A :: CCR7

C

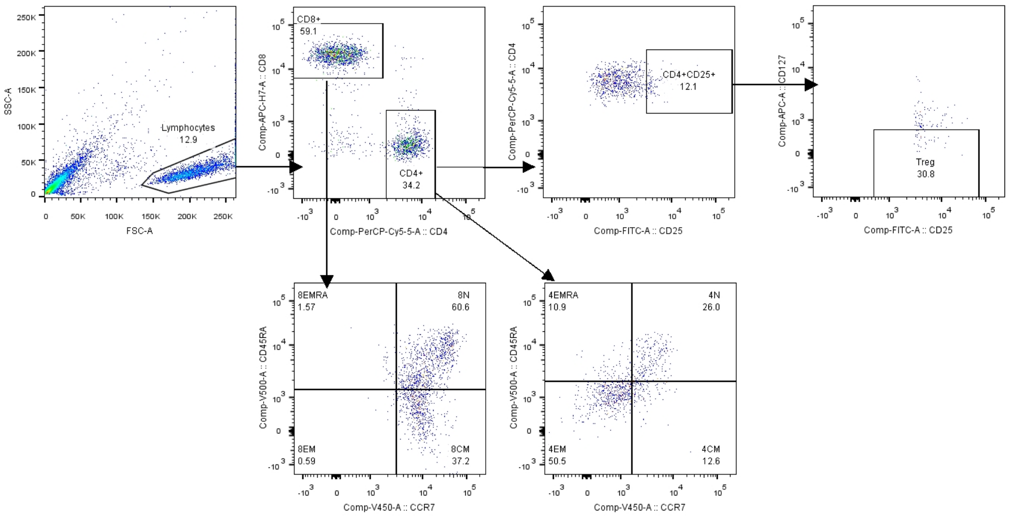
Lymphocytes
12.9
SSC-A
FSC-A
CD8+
59.1
CD4+
34.2
Comp-APC-H7-A :: CD8
Comp-PerCP-Cy5-5-A :: CD4
CD4+CD25+
12.1
Comp-FITC-A :: CD25
Treg
30.8
Comp-APC-A :: CD127
8EMRA
1.57
8N
60.6
8EM
0.59
8CM
37.2
4EMRA
10.9
4N
26.0
4EM
50.5
4CM
12.6
Comp-V500-A :: CD45RA
Comp-V450-A :: CCR7

Supplementary Figure 1. Gating strategy for identification of T cell subsets. (**A**) Gating strategy for identification of T lymphocytes from different samples (PB, 45RA+ and CB) at before culture. (**B**) Gating strategy for the identification of different T lymphocyte subsets (T_N, T_{CM}, T_{EM} and T_{EMRA}) in PB and CB samples before culture. (**C**) Gating strategy for identification of different T cell subpopulation after 14-day culture. CB, cord blood; PB, peripheral blood; 45RA, CD45RA+ fraction post-apheresis. Treg, Regulatory T lymphocytes; T_N, naïve T cell; T_{CM}, central memory T cells; T_{EM}, effector memory T cells; T_{EMRA}, terminal effector T cells.

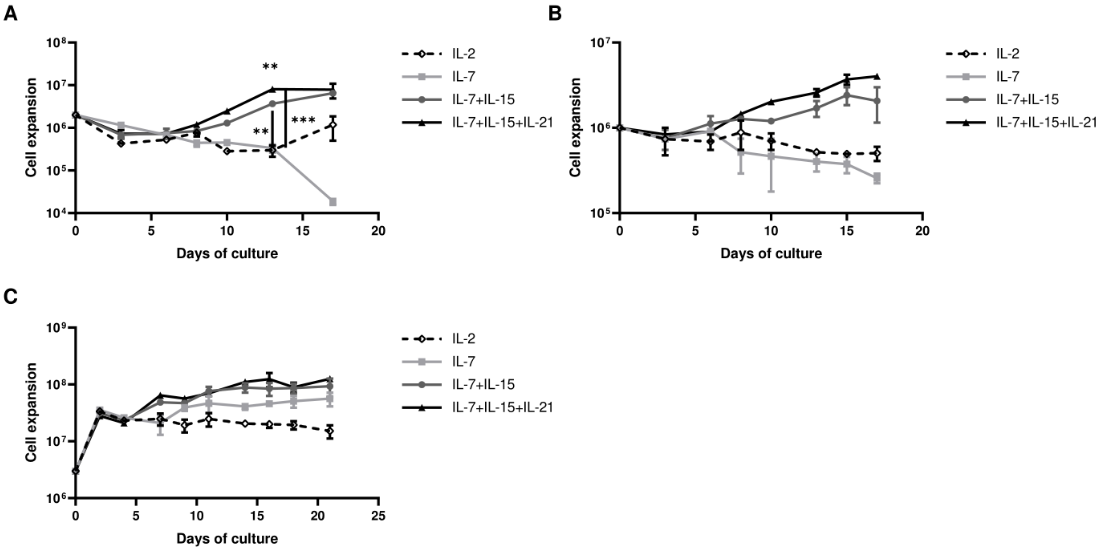

Supplementary Figure 2. Proliferation of T cells in culture under different cytokine conditions (IL-2, IL-7, IL-7+IL-15 or IL-7+IL-15+IL-21). (**A**) Proliferation of PB-derived lymphocytes during 17 days of culture. (**B**) Proliferation of 45RA+ post-apheresis-derived lymphocytes during 17 days of culture. (**C**) Proliferation of CB-derived lymphocytes during 21 days of culture. **p value < 0.005. ***p value < 0.0005. CB, cord blood; PB, peripheral blood.

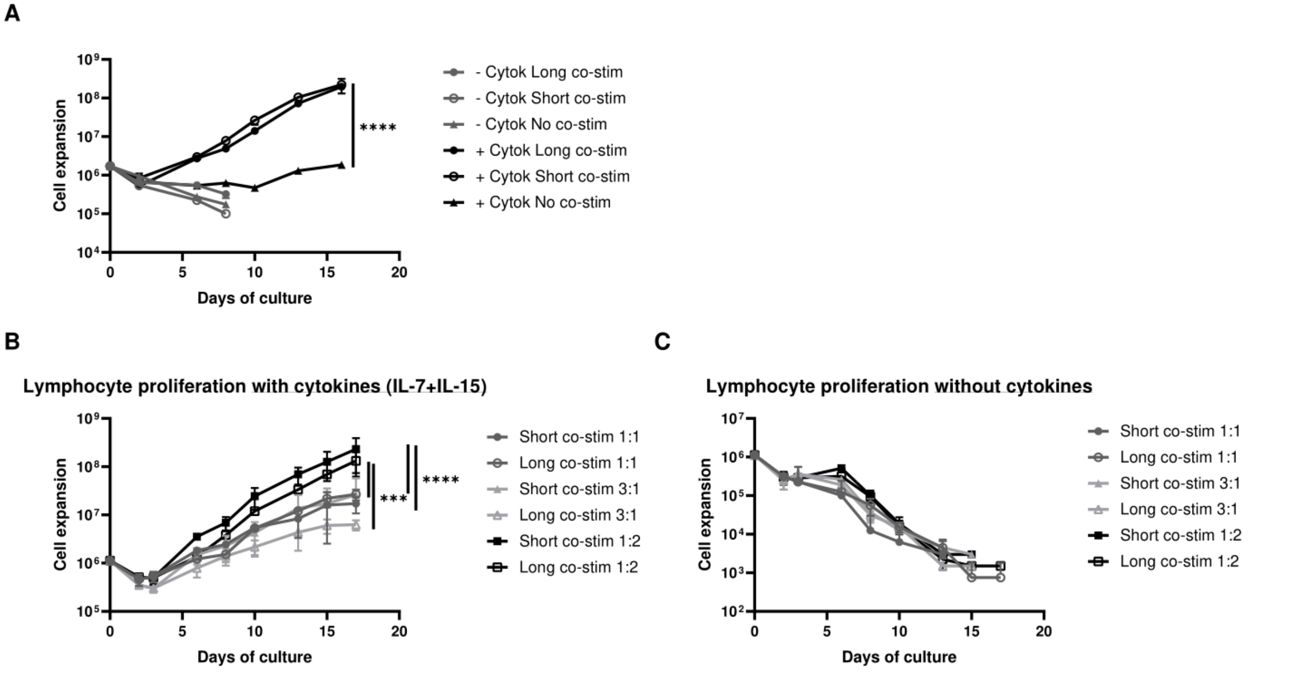

Supplementary Figure 3. Proliferation of peripheral blood T cells with or without beads and cytokines. (**A**) Lymphocyte proliferation in different culture conditions with or without cytokines and anti-CD3/CD28 co-stimulus. (**B**) Lymphocyte proliferation without cytokines and short (48 hours) or long (all culture period) co-stimulation with beads and different bead:T cell ratios (1:1, 3:1, 1:2). (**C**) Lymphocyte proliferation in culture with IL-7+IL-15 cytokines and short/long co-stimulation with beads at different bead:T cell ratios (1:1, 3:1, 1:2). ***p value < 0.0005. ****p value < 0.0001.

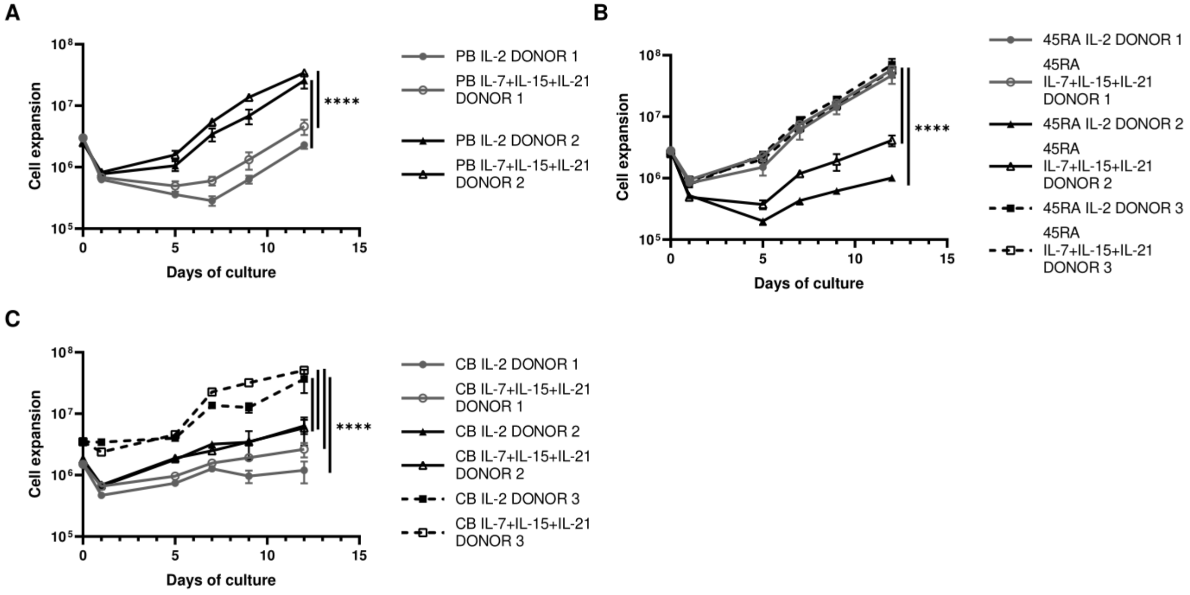

Supplementary Figure 4. Proliferation of lymphocytes form different donors cultured with IL-2 or IL-7+IL-15+IL-21. (**A**) Proliferation of PB-derived lymphocytes from 2 different healthy donors. (**B**)

Proliferation of 45RA+ T cells from 3 different donors. (**C**) Proliferation of CB-derived lymphocytes from 3 different healthy donors. ****p value < 0.0001. CB, cord blood; PB, peripheral blood; 45RA, CD45RA+ fraction post-apheresis.

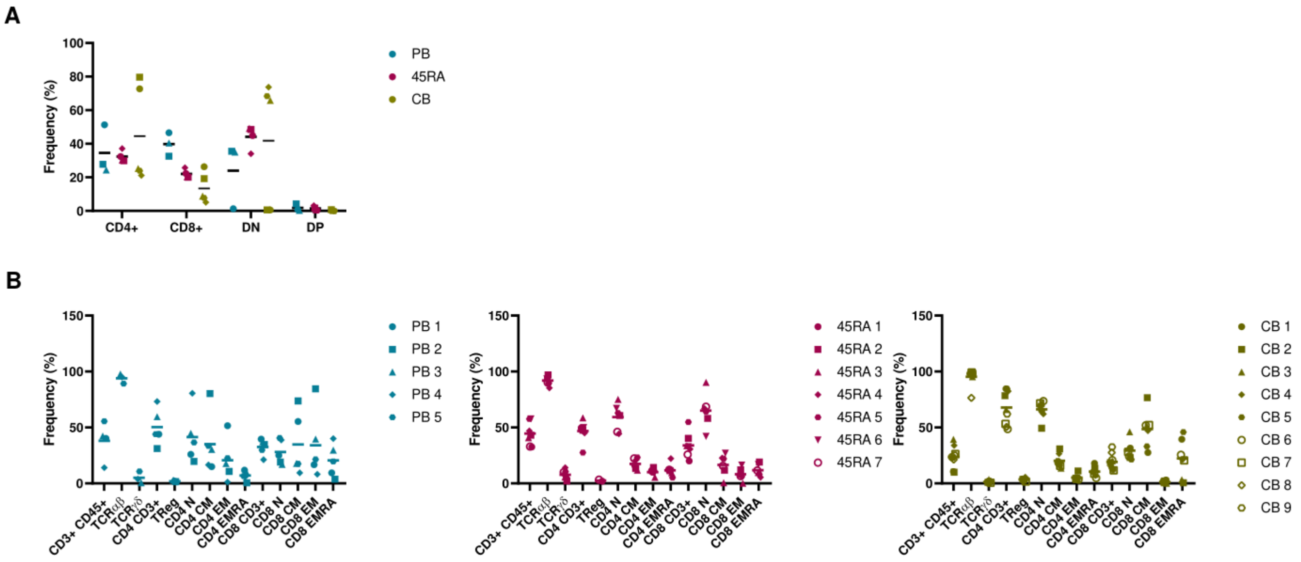

Supplementary Figure 5. Phenotype of T cells from PB, 45RA and CB before culture. (**A**) Cell phenotype according to CD4 and CD8 markers. DN: double negative (CD4-CD8-). DP: double positive (CD4+CD8+). (**B**) Different T cell subsets assessed by flow cytometry. CB, cord blood; PB, peripheral blood; 45RA, CD45RA+ fraction post-apheresis.

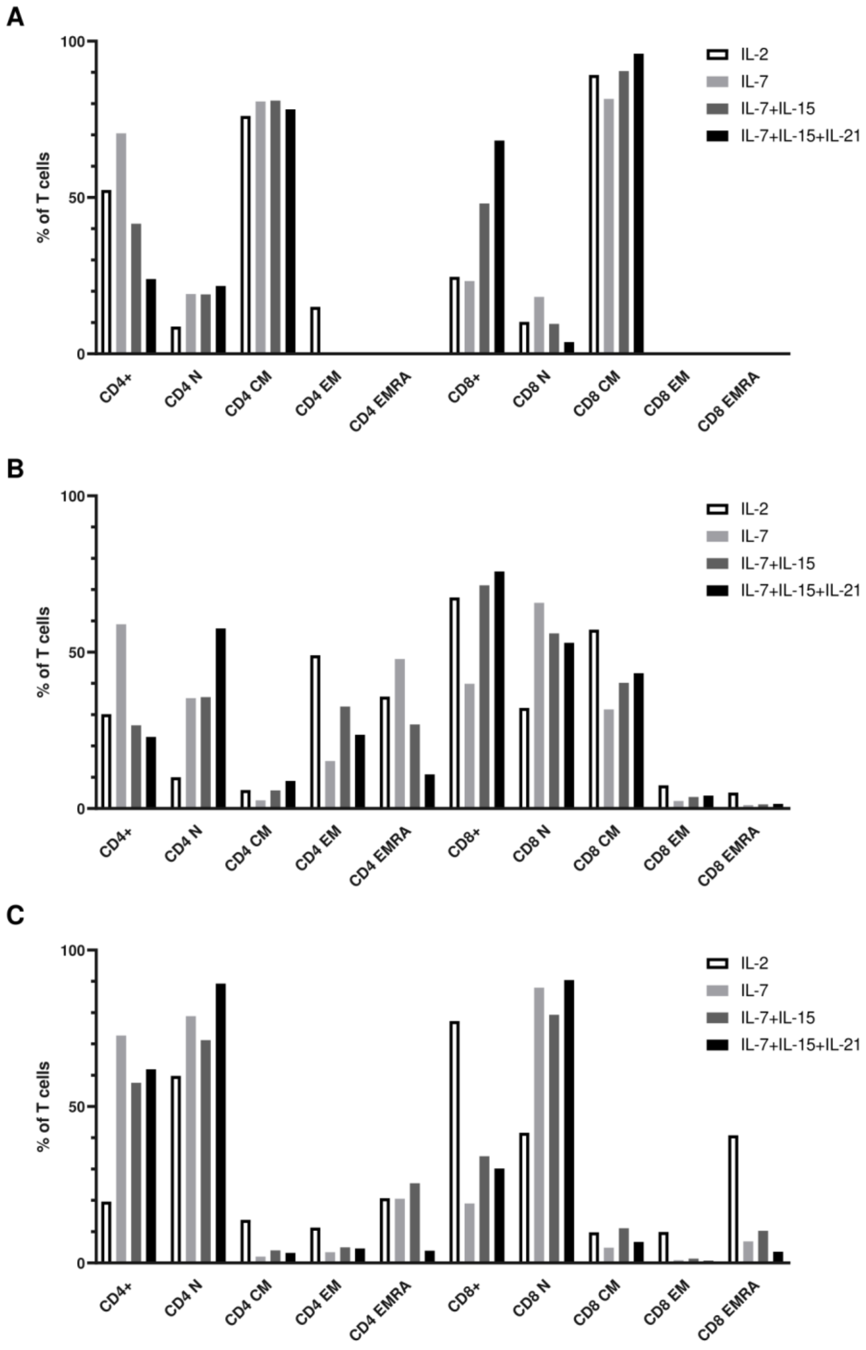

Supplementary Figure 6. Phenotype of T cell subsets in CD4+ and CD8+ lymphocytes after 10 days in culture under different cytokine conditions (IL-2, IL-7, IL-7+IL-15 or IL-7+IL-15+IL-21). (**A**) Phenotype of cells derived from PB. (**B**) Phenotype of T cells derived from 45RA fraction after apheresis. (**C**) Phenotype of T cells derived from CB. CB, cord blood; PB, peripheral blood; 45RA, CD45RA+ fraction post-apheresis.

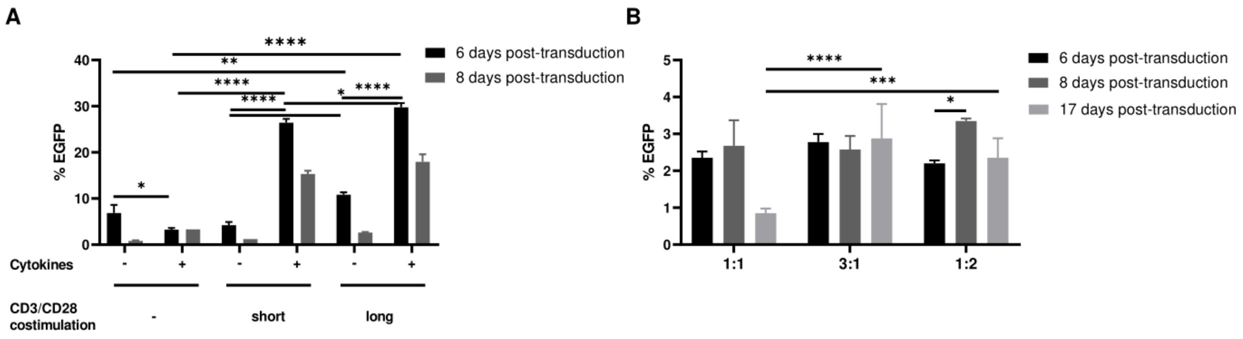

Supplementary Figure 7. Transduction efficiency of an EGFP-expressing LV in T cells in different culture conditions. (**A**) Lymphocyte transduction in culture with or without cytokines and beads co-stimulation. (**B**) Lymphocyte transduction in culture with different ratios (1:1, 3:1, 1:2) of beads co-stimulus. *p value < 0.05. **p value < 0.005. ***p value < 0.0005. ****p value < 0.0001.

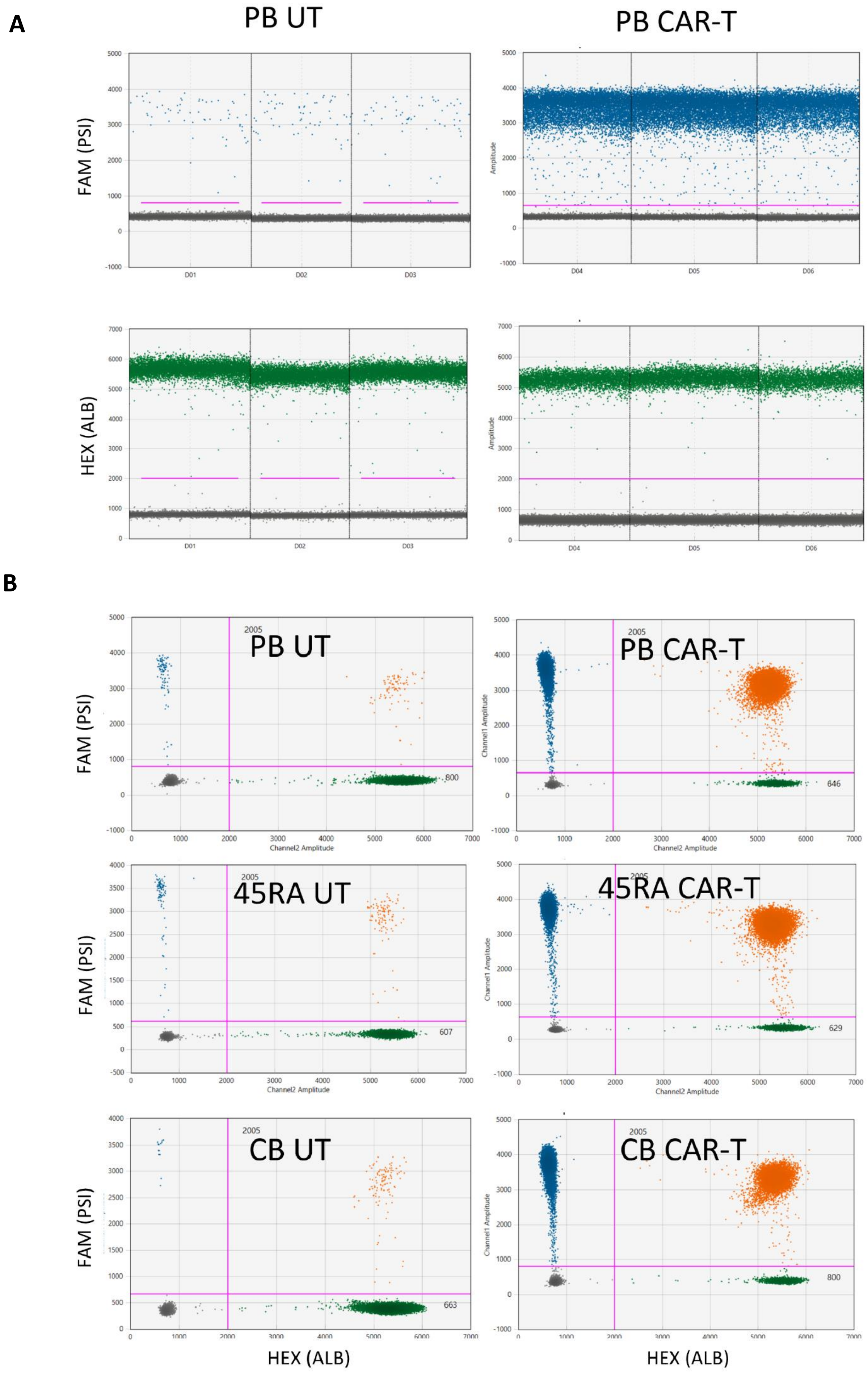
A
PB UT
PB CAR-T
FAM (PSI)
HEX (ALB)
Amplitude
D01
D02
D03
D04
D05
D06
B
PB UT
PB CAR-T
45RA UT
45RA CAR-T
CB UT
CB CAR-T
2005
800
646
607
629
663
800
Channel1 Amplitude
Channel2 Amplitude
FAM (PSI)
HEX (ALB)

Supplementary Figure 8. Vector copy number by ddPCR. (**A**) Spikes of Psi signal in FAM channel and albumin gene in HEX channel of untransduced PB T cells (PB UT) and PB CAR-T samples. (**B**) Representative data for thresholds set at 600 for psi signal (FAM channel in blue) and 2000 for albumin internal control (HEX channel in green) in 2D amplitude of CAR-T derived from PB, 45RA and CB, and the controls of untransduced T cells. CB, cord blood; PB, peripheral blood; 45RA, CD45RA+ fraction post-apheresis.

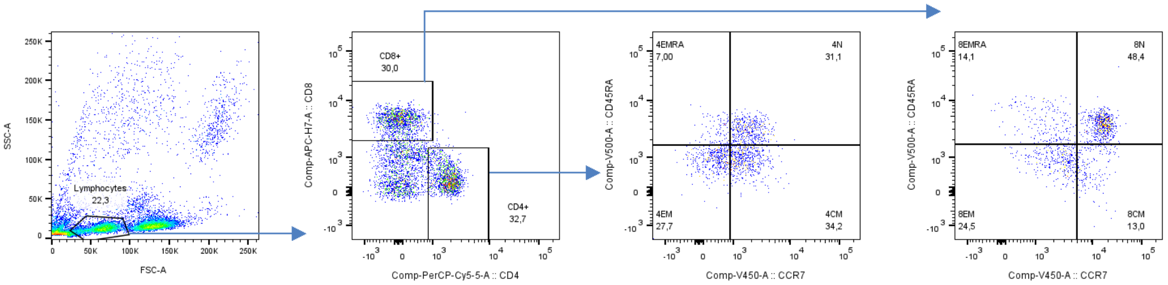

Supplementary Figure 9. PBMCs before treatment stained with antibodies for the identification of T lymphocyte subsets: naïve T cells (T_N), central memory T cells (T_{CM}), effector memory T cells (T_{EM}) and terminal effector T cells (T_{EMRA}).

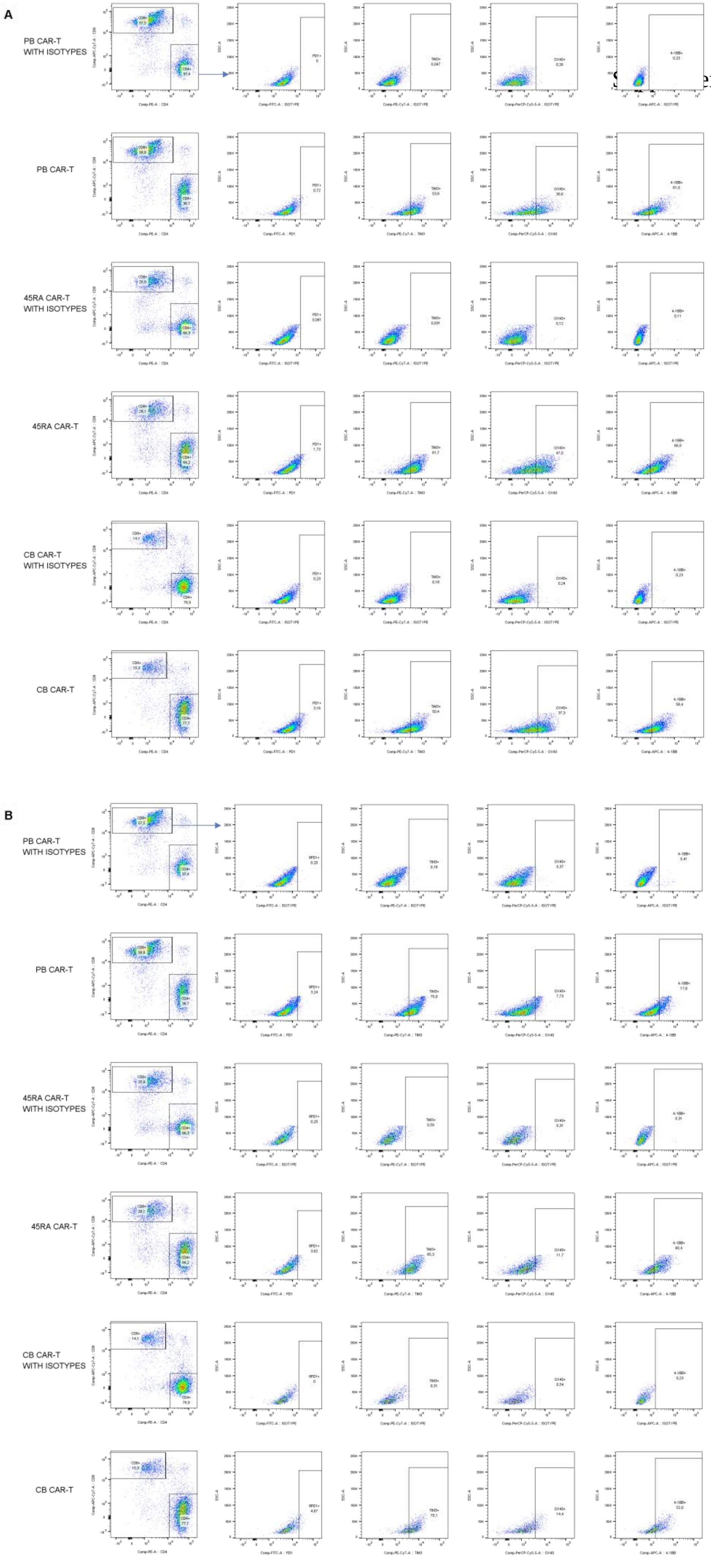
A
PB CAR-T
WITH ISOTYPES
PB CAR-T
45RA CAR-T
WITH ISOTYPES
45RA CAR-T
CB CAR-T
WITH ISOTYPES
CB CAR-T
B
PB CAR-T
WITH ISOTYPES
PB CAR-T
45RA CAR-T
WITH ISOTYPES
45RA CAR-T
CB CAR-T
WITH ISOTYPES
CB CAR-T

Supplementary Figure 10. Representative dot plots of exhaustion status of CAR-T cells prior co-cultures including controls with isotype stanning. (**A**) Dot plots of CD4+ CAR-T cells. (**B**) Dot plots of CD8+ CAR-T cells.

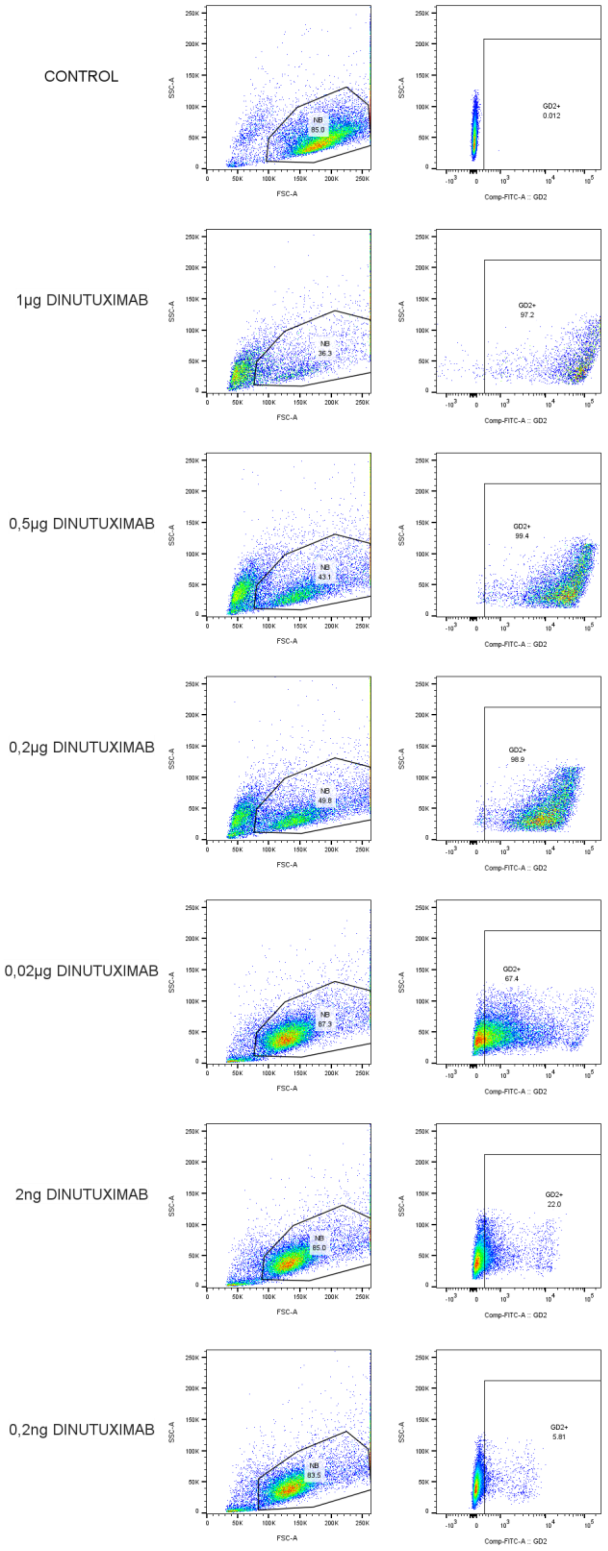

Supplementary Figure 11. Representative dot plots of LAN-1 cell line stained with different concentrations of Dinutuximab conjugated to FITC (1µg, 0.5µg, 0.2µg, 0.02µg, 2ng and 0.2ng).

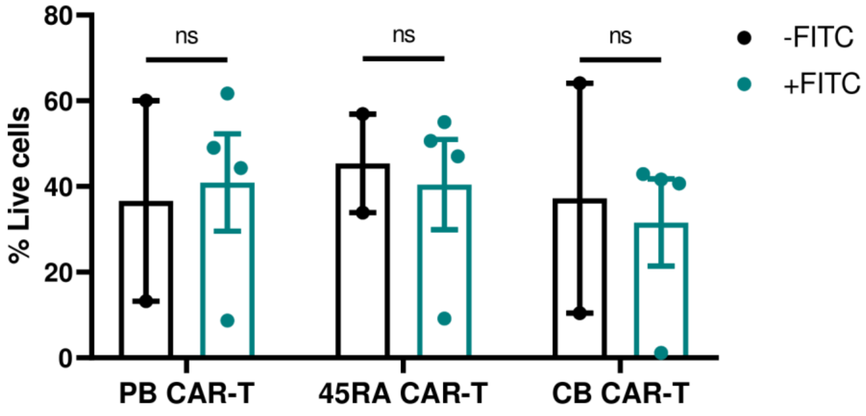

Supplementary Figure 12. LAN-1 cell viability (non-normalized) assessed by flow cytometry in co-culture with CAR-T cells at 2:1 CAR:NB ratio.

1.2 Supplementary Tables

Supplementary Table 1. MQR files of each sample processed for OGM (separate pdf document).

Supplementary Table 2. Structural variants (SVs) found in each sample (control samples at day 0 and CAR-T products at day 7) (separate excel document).
