# Supplementary material for: Choosing T-cell sources determines CAR-T cell activity in neuroblastoma
Source: Front Immunol. 2024 Mar 27;15:1375833. doi: 10.3389/fimmu.2024.1375833 (PMC11004344; doi:10.3389/fimmu.2024.1375833)
Supplement: Supplementary file 2 [file Table_1.pdf]

Job Details

label	value	description
Job ID	285,030	Job Identifier
Created at	2023-05-05T10:31:43.824Z	Date job was created
Updated at	2023-05-05T15:21:31.105Z	Date job was updated
User Name	Barbara Fernandez Garoz	Full name of user who launched job
Job type	Merge BNXs	The type of operation performed
Access Version	1.7.2	Bionano Access Version
Solve Version	Solve3.7_20221013_25	Bionano Solve Version
Compute On Demand Version	Solve3.7_20221013_150614_25	Version of pipeline for Compute On Demand
Job Name	PBC- - Molecule Merge	Alias for Job
Project Name	Tumores solidos	Name of the project
Sample Name	PBC-	Name of the sample
Sample UID	bef8006a-e5ae-11ed-bcb3-3cfdfe864a72	System generated global unique identifier
Reference	hg38_DLE1_0kb_0labels.cmap	Name of the reference genome this sample was aligned to

MQR Report Details

label	value	description
Version	1	Report version
Total DNA (≥ 150kbp)	5,065.99 Gbp	Total amount of DNA from molecules that are 150kbp or longer
N50 (≥ 150kbp)	249.75 kbp	N50 of DNA molecules that are 150kbp or longer
N50 (≥ 20 kbp)	183 kbp	N50 of the molecules that are 20kbp or longer)
Total DNA (≥ 20kbp)	8,342.48 Gbp	Total amount of DNA from molecules that are 20 kbp or longer
Total DNA (≥ 150kbp and min sites ≥ 9)	4,841.87 Gbp	Same as other Total DNA fields, but molecules must have at least 9 labels
N50 (≥ 150kbp and min sites ≥ 9)	251.25 kbp	Same as other N50 fields, but molecules must have at least 9 labels
Label color	BNGFLGR001	Label color used for detection.
Site	CTTAAG	Recognition sequence of the enzyme used.
Average label density (≥ 150kbp)	14.86 /100kbp	Average number of labels per 100 kbp for the molecules that are 150kbp or longer
Enzyme	DLE-1	Name of the enzyme used in this sample.
Reference	hg38_DLE1_0kb_0labels.cmap	Name of the reference genome this sample was aligned to.
Reference Length	3,088,269,832 bp	Total length of reference sequence
Effective coverage	1,359.3	Total amount of aligned DNA divided by the size of the reference genome.
Map rate	86.7 %	Percentage of molecules that are 150kbp or longer mapped to the reference
Scaling SD	0	Linear term in sizing error relative to reference
Site SD	0.096	Constant term in sizing error relative to reference

label	value	description
Relative SD	0.016	Quadratic term in sizing error relative to reference
Base pairs per pixel	464.97	Calculated base pairs per pixel in the alignment by comparing molecules to the reference.
Positive label variance (PLV)	2.98	Percentage of labels absent in reference
Negative label variance (NLV)	7.05	Percentage of reference labels absent in molecules
Molecule integrity number	0.13	Measure of molecule quality

Job Details

label	value	description
Job ID	285,031	Job Identifier
Created at	2023-05-05T10:32:31.669Z	Date job was created
Updated at	2023-05-05T16:36:36.426Z	Date job was updated
User Name	Barbara Fernandez Garoz	Full name of user who launched job
Job type	Merge BNXs	The type of operation performed
Access Version	1.7.2	Bionano Access Version
Solve Version	Solve3.7_20221013_25	Bionano Solve Version
Compute On Demand Version	Solve3.7_20221013_150614_25	Version of pipeline for Compute On Demand
Job Name	PBCART - Molecule Merge	Alias for Job
Project Name	Tumores solidos	Name of the project
Sample Name	PBCART	Name of the sample
Sample UID	d529bf68-e5ae-11ed-bcb3-3cfdfe864a72	System generated global unique identifier
Reference	hg38_DLE1_0kb_0labels.cmap	Name of the reference genome this sample was aligned to

MQR Report Details

label	value	description
Version	1	Report version
Total DNA (≥ 150kbp)	5,015.59 Gbp	Total amount of DNA from molecules that are 150kbp or longer
N50 (≥ 150kbp)	214.5 kbp	N50 of DNA molecules that are 150kbp or longer
N50 (≥ 20 kbp)	139.75 kbp	N50 of the molecules that are 20kbp or longer)
Total DNA (≥ 20kbp)	11,064.39 Gbp	Total amount of DNA from molecules that are 20 kbp or longer
Total DNA (≥ 150kbp and min sites ≥ 9)	4,811.57 Gbp	Same as other Total DNA fields, but molecules must have at least 9 labels
N50 (≥ 150kbp and min sites ≥ 9)	215.47 kbp	Same as other N50 fields, but molecules must have at least 9 labels
Label color	BNGFLGR001	Label color used for detection.
Site	CTTAAG	Recognition sequence of the enzyme used.
Average label density (≥ 150kbp)	15.1 /100kbp	Average number of labels per 100 kbp for the molecules that are 150kbp or longer
Enzyme	DLE-1	Name of the enzyme used in this sample.
Reference	hg38_DLE1_0kb_0labels.cmap	Name of the reference genome this sample was aligned to.
Reference Length	3,088,269,832 bp	Total length of reference sequence
Effective coverage	1,304.06	Total amount of aligned DNA divided by the size of the reference genome.
Map rate	83.7 %	Percentage of molecules that are 150kbp or longer mapped to the reference
Scaling SD	0	Linear term in sizing error relative to reference
Site SD	0.094	Constant term in sizing error relative to reference

label	value	description
Relative SD	0.021	Quadratic term in sizing error relative to reference
Base pairs per pixel	481.88	Calculated base pairs per pixel in the alignment by comparing molecules to the reference.
Positive label variance (PLV)	2.86	Percentage of labels absent in reference
Negative label variance (NLV)	6.22	Percentage of reference labels absent in molecules
Molecule integrity number	0.13	Measure of molecule quality

Job Details

label	value	description
Job ID	94,482	Job Identifier
Created at	2023-12-12T14:37:35.548Z	Date job was created
Updated at	2023-12-12T19:52:16.444Z	Date job was updated
User Name	Barbara Fernandez	Full name of user who launched job
Job type	Merge BNXs	The type of operation performed
Access Version	1.8	Bionano Access Version
Solve Version	Solve3.8	Bionano Solve Version
Compute On Demand Version	Solve3.8	Version of pipeline for Compute On Demand
Job Name	45 RA Co - - Molecule Merge	Alias for Job
Project Name	Investigacion	Name of the project
Sample Name	45 RA Co -	Name of the sample
Sample UID	ffa56c6a-69bb-11ee-bbf4-3cfdfe97c430	System generated global unique identifier
Reference	hg38_DLE1_0kb_0labels_masked_YPARs.cmap	Name of the reference genome this sample was aligned to

MQR Report Details

label	value	description
Version	1	Report version
Total DNA (≥ 150kbp)	4,153.07 Gbp	Total amount of DNA from molecules that are 150kbp or longer
N50 (≥ 150kbp)	225 kbp	N50 of DNA molecules that are 150kbp or longer
N50 (≥ 20 kbp)	130.13 kbp	N50 of the molecules that are 20kbp or longer)
Total DNA (≥ 20kbp)	9,810.53 Gbp	Total amount of DNA from molecules that are 20 kbp or longer
Total DNA (≥ 150kbp and min sites ≥ 9)	4,036.66 Gbp	Same as other Total DNA fields, but molecules must have at least 9 labels
N50 (≥ 150kbp and min sites ≥ 9)	226.5 kbp	Same as other N50 fields, but molecules must have at least 9 labels
Label color	BNGFLGR001	Label color used for detection.
Site	CTTAAG	Recognition sequence of the enzyme used.
Average label density (≥ 150kbp)	16.6 /100kbp	Average number of labels per 100 kbp for the molecules that are 150kbp or longer
Enzyme	DLE-1	Name of the enzyme used in this sample.
Reference	hg38_DLE1_0kb_0labels_masked_YPARs.cmap	Name of the reference genome this sample was aligned to.
Reference Length	3,088,269,832 bp	Total length of reference sequence

label	value	description
Effective coverage	903.2	Total amount of aligned DNA divided by the size of the reference genome.
Map rate	69.1 %	Percentage of molecules that are 150kbp or longer mapped to the reference
Scaling SD	0.017	Linear term in sizing error relative to reference
Site SD	0.1	Constant term in sizing error relative to reference
Relative SD	0.02	Quadratic term in sizing error relative to reference
Base pairs per pixel	489.07	Calculated base pairs per pixel in the alignment by comparing molecules to the reference.
Positive label variance (PLV)	6.13	Percentage of labels absent in reference
Negative label variance (NLV)	7.36	Percentage of reference labels absent in molecules
Molecule integrity number	0.23	Measure of molecule quality

Job Details

label	value	description
Job ID	94,483	Job Identifier
Created at	2023-12-12T14:41:39.268Z	Date job was created
Updated at	2023-12-12T21:01:20.961Z	Date job was updated
User Name	Barbara Fernandez	Full name of user who launched job
Job type	Merge BNXs	The type of operation performed
Access Version	1.8	Bionano Access Version
Solve Version	Solve3.8	Bionano Solve Version
Compute On Demand Version	Solve3.8	Version of pipeline for Compute On Demand
Job Name	45 RA CAR T - Molecule Merge	Alias for Job
Project Name	Investigacion	Name of the project
Sample Name	45 RA CAR T	Name of the sample
Sample UID	20206b98-69bc-11ee-a1b0-3cfdfe97c430	System generated global unique identifier
Reference	hg38_DLE1_0kb_0labels_masked_YPARs.cmap	Name of the reference genome this sample was aligned to

MQR Report Details

label	value	description
Version	1	Report version
Total DNA (≥ 150kbp)	4,336.69 Gbp	Total amount of DNA from molecules that are 150kbp or longer
N50 (≥ 150kbp)	219.75 kbp	N50 of DNA molecules that are 150kbp or longer
N50 (≥ 20 kbp)	116.25 kbp	N50 of the molecules that are 20kbp or longer)
Total DNA (≥ 20kbp)	12,113.37 Gbp	Total amount of DNA from molecules that are 20 kbp or longer
Total DNA (≥ 150kbp and min sites ≥ 9)	4,117.95 Gbp	Same as other Total DNA fields, but molecules must have at least 9 labels
N50 (≥ 150kbp and min sites ≥ 9)	219.38 kbp	Same as other N50 fields, but molecules must have at least 9 labels
Label color	BNGFLGR001	Label color used for detection.
Site	CTTAAG	Recognition sequence of the enzyme used.
Average label density (≥ 150kbp)	19.13 /100kbp	Average number of labels per 100 kbp for the molecules that are 150kbp or longer
Enzyme	DLE-1	Name of the enzyme used in this sample.
Reference	hg38_DLE1_0kb_0labels_masked_YPARs.cmap	Name of the reference genome this sample was aligned to.
Reference Length	3,088,269,832 bp	Total length of reference sequence

label	value	description
Effective coverage	820.05	Total amount of aligned DNA divided by the size of the reference genome.
Map rate	61.5 %	Percentage of molecules that are 150kbp or longer mapped to the reference
Scaling SD	0.021	Linear term in sizing error relative to reference
Site SD	0.11	Constant term in sizing error relative to reference
Relative SD	0.016	Quadratic term in sizing error relative to reference
Base pairs per pixel	476.43	Calculated base pairs per pixel in the alignment by comparing molecules to the reference.
Positive label variance (PLV)	5.25	Percentage of labels absent in reference
Negative label variance (NLV)	8.87	Percentage of reference labels absent in molecules
Molecule integrity number	0.13	Measure of molecule quality

Job Details

label	value	description
Job ID	40,951	Job Identifier
Created at	2023-05-23T12:22:12.131Z	Date job was created
User Name	Barbara Fernandez	Full name of user who launched job
Job type	Merge BNXs	The type of operation performed
Access Version	1.7.2	Bionano Access Version
Solve Version	Solve3.7_20221013_25	Bionano Solve Version
Compute On Demand Version	Solve3.7_20221013_150614_25	Version of pipeline for Compute On Demand
Job Name	CBCO- - Molecule Merge	Alias for Job
Project Name	Investigacion	Name of the project
Sample Name	CBCO-	Name of the sample
Sample UID	f0704afe-ed91-11ed-9faf-3cfdfe97c430	System generated global unique identifier
Reference	hg38_DLE1_0kb_0labels.cmap	Name of the reference genome this sample was aligned to

MQR Report Details

label	value	description
Reference	hg38_DLE1_0kb_0labels.cmap	Name of the reference genome this sample was aligned to.
Reference Length	3,088,269,832 bp	Total length of reference sequence
Enzyme	DLE-1	Name of the enzyme used in this sample.
Site	CTTAAG	Recognition sequence of the enzyme used.
N50 (>= 20 kbp)	147 kbp	N50 of the molecules that are 20kbp or longer)
Total DNA (>= 20kbp)	10,279.09 Gbp	Total amount of DNA from molecules that are 20 kbp or longer
N50 (>= 150kbp)	237.75 kbp	N50 of DNA molecules that are 150kbp or longer
Total DNA (>= 150kbp)	5,031.34 Gbp	Total amount of DNA from molecules that are 150kbp or longer
N50 (>= 150kbp and min sites >=9)	239.25 kbp	Same as other N50 fields, but molecules must have at least 9 labels
Total DNA (>= 150kbp and min sites >= 9)	4,840.3 Gbp	Same as other Total DNA fields, but molecules must have at least 9 labels
Map rate	85.4 %	Percentage of molecules that are 150kbp or longer mapped to the reference
Effective coverage	1,338.49	Total amount of aligned DNA divided by the size of the reference genome times the map rate.
Average label density (>= 150kbp)	15.23 /100kbp	Average number of labels per 100 kbp for the molecules that are 150kbp or longer
Site SD	0.11	Constant term in sizing error relative to reference
Relative SD	0.018	Quadratic term in sizing error relative to reference
Scaling SD	0.013	Linear term in sizing error relative to reference
integrity_num	0.11	

label	value	description
Negative label variance (NLV)	8.94	Percentage of reference labels absent in molecules
Base pairs per pixel	482.68	Calculated base pairs per pixel in the alignment by comparing molecules to the reference.
Label color	BNGFLGR001	Label color used for detection.
version	1	
Positive label variance (PLV)	3.48	Percentage of labels absent in reference

Job Details

label	value	description
Job ID	40,952	Job Identifier
Created at	2023-05-23T12:23:14.353Z	Date job was created
User Name	Barbara Fernandez	Full name of user who launched job
Job type	Merge BNXs	The type of operation performed
Access Version	1.7.2	Bionano Access Version
Solve Version	Solve3.7_20221013_25	Bionano Solve Version
Compute On Demand Version	Solve3.7_20221013_150614_25	Version of pipeline for Compute On Demand
Job Name	CBCART - Molecule Merge	Alias for Job
Project Name	Investigacion	Name of the project
Sample Name	CBCART	Name of the sample
Sample UID	dc52edb0-ed91-11ed-9e59-3cfdfe97c430	System generated global unique identifier
Reference	hg38_DLE1_0kb_0labels.cmap	Name of the reference genome this sample was aligned to

MQR Report Details

label	value	description
Reference	hg38_DLE1_0kb_0labels.cmap	Name of the reference genome this sample was aligned to.
Reference Length	3,088,269,832 bp	Total length of reference sequence
Enzyme	DLE-1	Name of the enzyme used in this sample.
Site	CTTAAG	Recognition sequence of the enzyme used.
N50 (>= 20 kbp)	131.63 kbp	N50 of the molecules that are 20kbp or longer)
Total DNA (>= 20kbp)	10,860.13 Gbp	Total amount of DNA from molecules that are 20 kbp or longer
N50 (>= 150kbp)	226.88 kbp	N50 of DNA molecules that are 150kbp or longer
Total DNA (>= 150kbp)	4,673.41 Gbp	Total amount of DNA from molecules that are 150kbp or longer
N50 (>= 150kbp and min sites >=9)	228 kbp	Same as other N50 fields, but molecules must have at least 9 labels
Total DNA (>= 150kbp and min sites >= 9)	4,545.26 Gbp	Same as other Total DNA fields, but molecules must have at least 9 labels
Map rate	70.3 %	Percentage of molecules that are 150kbp or longer mapped to the reference
Effective coverage	1,034.66	Total amount of aligned DNA divided by the size of the reference genome times the map rate.
Average label density (>= 150kbp)	16.09 /100kbp	Average number of labels per 100 kbp for the molecules that are 150kbp or longer
Site SD	0.12	Constant term in sizing error relative to reference
Relative SD	0.025	Quadratic term in sizing error relative to reference
Scaling SD	0	Linear term in sizing error relative to reference
integrity_num	0.18	

label	value	description
Negative label variance (NLV)	11.54	Percentage of reference labels absent in molecules
Base pairs per pixel	489.44	Calculated base pairs per pixel in the alignment by comparing molecules to the reference.
Label color	BNGFLGR001	Label color used for detection.
version	1	
Positive label variance (PLV)	4	Percentage of labels absent in reference
